# Crosslinking by ZapD drives the assembly of short FtsZ filaments into toroidal structures in solution

**DOI:** 10.7554/eLife.95557

**Published:** 2025-09-15

**Authors:** Adrián Merino-Salomón, Jonathan Schneider, Leon Babl, Jan-Hagen Krohn, Marta Sobrinos-Sanguino, Tillman Schäfer, Juan Ramon Luque-Ortega, Carlos Alfonso, Mercedes Jiménez, Marion Jasnin, Petra Schwille, Germán Rivas

**Affiliations:** 1 Department of Cellular and Molecular Biophysics, Max Planck Institute of Biochemistry Martinsried Germany; 2 Department of Molecular Structural Biology, Max Planck Institute of Biochemistry Martinsried Germany; 3 Exzellenzcluster ORIGINS Garching Germany; 4 Molecular Interactions Facility, Centro de Investigaciones Biológicas Margarita Salas, Consejo Superior de Investigaciones Científicas (CSIC) Madrid Spain; 5 Cryo-EM Facility, Max Planck Institute of Biochemistry Martinsried Germany; 6 Centro de Investigaciones Biológicas Margarita Salas, Consejo Superior de Investigaciones Científicas (CSIC) Madrid Spain; 7 Helmholtz Pioneer Campus, Helmholtz Munich Neuherberg Germany; 8 Department of Chemistry, Technical University of Munich Garching Germany; University of Warwick United Kingdom; Universitat Pompeu Fabra Spain

**Keywords:** bacterial division, FtsZ, ZapD, divisome, *E. coli*, toroid, *E. coli*

## Abstract

Cell division in *Escherichia coli* relies on the Z ring, a cytoskeletal structure that acts as a scaffold for the assembly of the divisome. To date, the detailed mechanisms underlying the assembly and stabilization of the Z ring remain elusive. This study highlights the role of the FtsZ-associated protein (Zap) ZapD in the assembly and stabilization of Z-ring-like structures via filament crosslinking. Using cryo-electron tomography and biochemical analysis, we show that, at equimolar concentrations of ZapD and FtsZ, ZapD induces the formation of toroidal structures composed of short, curved FtsZ filaments that are crosslinked vertically, but also laterally and diagonally. At higher concentrations of ZapD, regularly spaced ZapD dimers crosslink FtsZ filaments from above, resulting in the formation of straight bundles. Despite the simplicity of this reconstituted system, these findings provide valuable insights into the structural organization and stabilization of the Z ring by Zap proteins in bacterial cells, revealing the key role of optimal crosslinking density and geometry in enabling filament curvature and ring formation.

## Introduction

Cell division in bacteria is a well-orchestrated process mediated by a multiprotein complex called the divisome. Their components interact reversibly to form a division ring, which is essential for cytokinesis (Levin and Janakiraman, 2021; Du and Lutkenhaus, 2019; Attaibi and den Blaauwen, 2022). At the core of this process in most bacteria is FtsZ, a tubulin homolog that polymerizes in the presence of GTP beneath the inner membrane at the future division site, providing a scaffold for recruiting other division proteins (Nogales et al., 1998; de Boer et al., 1992; Addinall and Lutkenhaus, 1996; McQuillen and Xiao, 2020; Wang et al., 2020). FtsZ undergoes treadmilling due to polymerization-dependent GTP hydrolysis, allowing the ring to exhibit its dynamic behavior (Wagstaff et al., 2017). Depending on environmental conditions, FtsZ filaments can assemble into various structures, including bundles, sheets, and toroids (Lu et al., 2000; Mukherjee and Lutkenhaus, 1999; Löwe and Amos, 1999; González et al., 2003; Popp et al., 2009). Membrane-tethered FtsZ assemblies are arranged as loosely structured spirals in the midcell region, guided by spatial regulators. These structures eventually coalesce to form a cohesive Z ring (Squyres et al., 2021).

**Figure 1.**
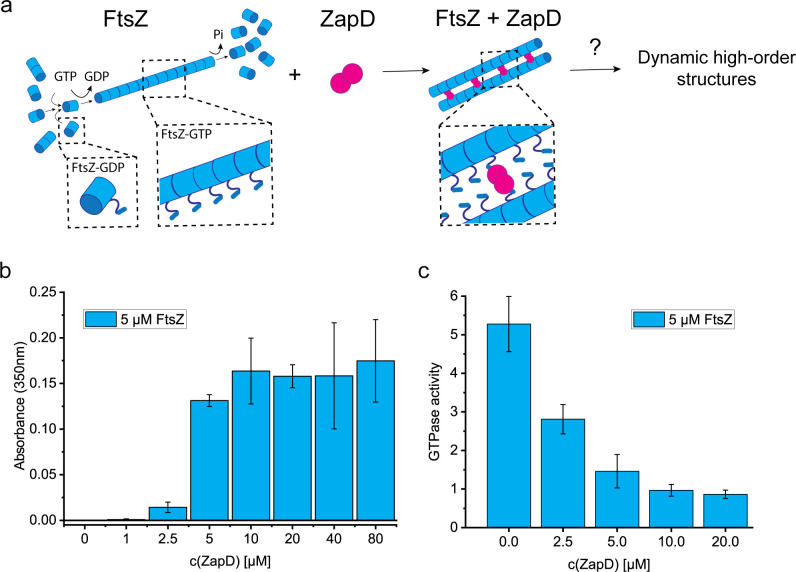
ZapD binds FtsZ and promotes filament bundling. (**a**) Scheme of the FtsZ protein and its interaction with ZapD. *E. coli* FtsZ (blue) monomers in solution oligomerize depending on the buffer conditions. Upon GTP binding, FtsZ homopolymerizes directionally, assembling single-stranded filaments. ZapD is a robust dimer (magenta) that interacts directly with FtsZ, crosslinking filaments by promoting lateral interactions between them. Although the molecular mechanism is still unclear, the current hypothesis of interaction assumes dimers of ZapD crosslinking two FtsZ filaments through the CCTP of FtsZ, expecting at around 1:1 (FtsZ:ZapD) molar ratio in a homogeneous bundle (1 dimer of ZapD connected to 2 monomers of FtsZ). According to this model, the orientation of the FtsZ filaments could be parallel or antiparallel, allowing the growth and treadmilling of the filaments. However, the mechanism of assembly of dynamic high-order structures is still unknown. (**b**) Turbidity assays measuring the absorbance at 350 nm of samples containing 5 µM FtsZ and increasing concentrations of ZapD. The turbidity of the sample was measured 5 min after the addition of 1 mM GTP at working buffer (50 mM KCl, 50 mM Tris-Cl, 5 mM MgCl_2_, pH 7). FtsZ polymers do not show a significant turbidity at this wavelength; therefore, the signal at 350 nm corresponds to the presence of large FtsZ macrostructures and bundles. The mean value and SD of >3 independent replicates are plotted in the graph. (**c**) GTPase activity of FtsZ after the addition of 1 mM GTP in the presence of increasing concentrations of ZapD at working conditions (50 mM KCl, 50 mM Tris-Cl, 5 mM MgCl_2_, pH 7). The mean value and SD plotted in the graph are the result of 3 independent replicates. The GTPase activity was measured as a result of the Pi released from GTP consumption. The units are mol GTP consumed per mol FtsZ per min.

The molecular components involved in cell division must be in the right place at the right time during the cell cycle. Several factors in this spatiotemporal regulation modulate FtsZ assembly. In *Escherichia coli*, FtsA and ZipA tether FtsZ to the membrane (Ma and Margolin, 1999; Hale and Boer, 2002; Wang et al., 1997) and, together with a set of FtsZ-associated proteins (Zaps), stabilize the ring (Durand-Heredia et al., 2012; Huang et al., 2013; Durand-Heredia et al., 2011; Ebersbach et al., 2008). Conversely, negative modulators inhibit FtsZ assembly at other sites, namely Min proteins at the polar regions (de Boer et al., 1989) and SlmA on the nucleoid (Tonthat et al., 2011). Most of these modulators interact with FtsZ through its carboxy-terminal end (CCTP), which modulates division assembly as a central hub (Ortiz et al., 2016; Buske and Levin, 2012). ZapD is the only Zap protein known to crosslink FtsZ by binding CCTP, suggesting a critical Z ring structure stabilizing function (Durand-Heredia et al., 2012; Schumacher et al., 2016; Huang et al., 2016; Choi et al., 2016; Roach et al., 2016; Figure 1a).

**Figure 1—figure supplement 1.**
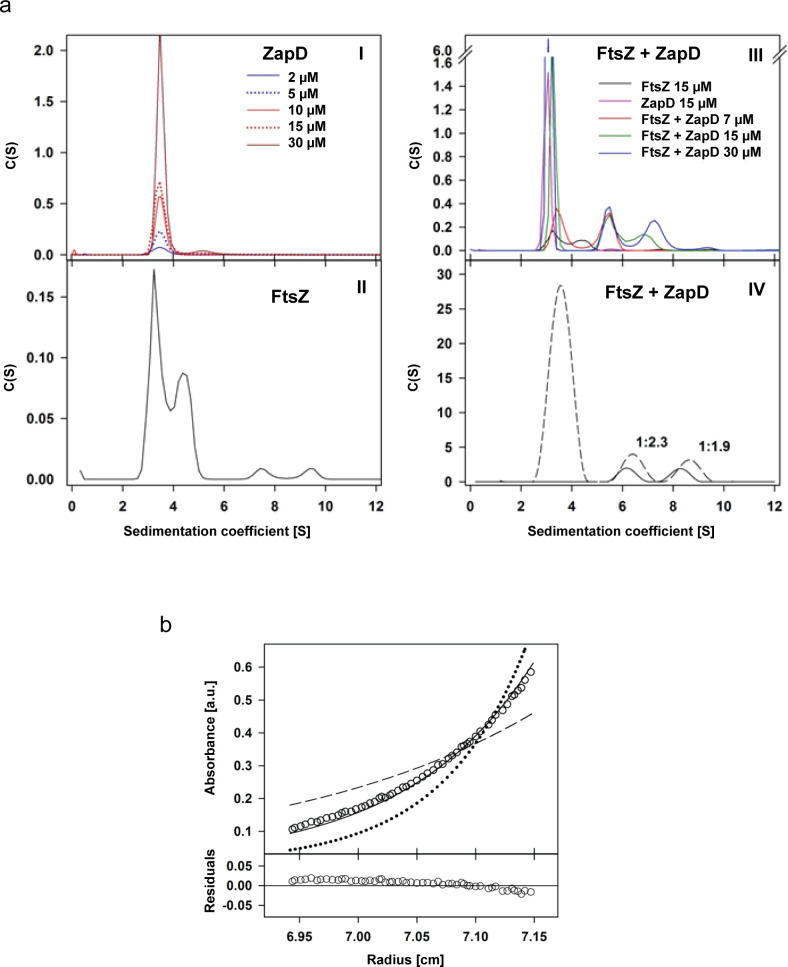
Characterization of the ZapD dimer and ZapD-FtsZ-GDP interaction by analytical ultracentrifugation (AUC*).* (**a**) Sedimentation coefficient distribution, *c*(*s*), obtained by sedimentation velocity (SV) with (**I**) ZapD at different protein concentrations at working conditions (50 mM KCl, 50 mM Tris-Cl, 5 mM MgCl_2_, pH 7). More than 95% of the sample sedimented as single species with an experimental sedimentation coefficient of 3.5 S once corrected to standard conditions (s20, w: 3.6 S±0.1). It matched with the expected value for ZapD dimers. (II) Sedimentation coefficient distribution, *c*(*s*), obtained by SV of FtsZ at 15 µM at working conditions (50 mM KCl, 50 mM Tris-Cl, 5 mM MgCl_2_, pH 7). (III) Sedimentation coefficient distribution, c(*s*), obtained by SV of the interaction of 15 µM FtsZ and increasing concentrations of ZapD in physiological glutamate-acetate buffer. There is an axis break at 1.6 to highlight protein complexes. (IV) Represents the global multi-wavelength (280 and 250 nm) analysis of the sedimentation coefficient distribution for FtsZ-ZapD complexes obtained by SV and decomposition into component sedimentation coefficient distributions *c*_*k*_(*s*) for FtsZ (solid trace) and ZapD (dashed trace) using FtsZ:ZapD initial ratio of 1:4. Numbers over the peaks correspond to the FtsZ:ZapD molar stoichiometry observed for each complex. (**b**) Concentration gradient obtained by sedimentation equilibrium (SE) of ZapD at 10 µM. Experimental data (empty circles) are shown together with the best-fit analysis corresponding to ZapD dimer (solid line), monomer (dashed line), and trimer (dotted line). The lower plot represents the residuals of the fitting of the experimental data to the dimeric model. ZapD was shown as a stable dimer, showing a buoyant mass of 14,415 Da, corresponding to a molecular mass of 56,200±400 Da, considering the partial specific volume calculated from the ZapD amino acid sequence.

**Figure 1—figure supplement 2.**
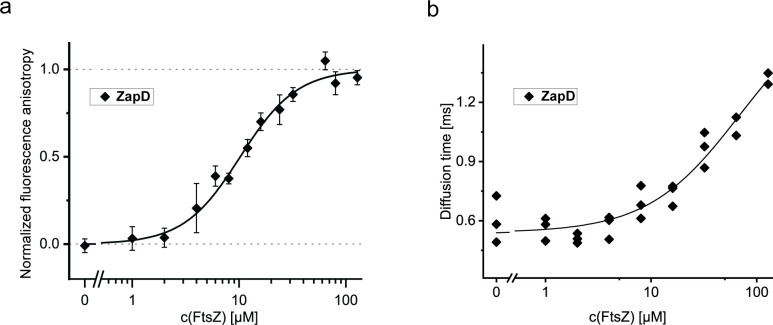
Biochemical characterization of the ZapD-FtsZ-GDP interaction by fluorescence correlation microscopy (FCS) and fluorescence anisotropy. (**a**) Interaction curve of FtsZ-GDP and ZapD by fluorescence anisotropy. The fluorescence anisotropy measurement of 5 µM ZapD supplemented with 150 nM ZapD-ATTO 647 N at increasing concentrations of FtsZ demonstrated a direct interaction between both proteins with an apparent *Kd* of 10.26±1.07 (SE). Samples were in working conditions (50 mM KCl, 50 mM Tris-Cl, 5 mM MgCl_2_, pH 7) and the error bars represent the standard deviation among 3 independent samples. (**b**) FCS analysis of increasing concentrations of FtsZ-GDP with 5 µM of ZapD supplemented with 100 nM ZapD-ATTO 647 N in working conditions. The FCS analysis demonstrated an increasing diffusion time of ZapD along with the FtsZ concentration as a result of higher proportion of ZapD bound to FtsZ. Negative controls were measured with either FtsZ or ZapD alone. The plotted line is only to guide the eye. Each condition was measured in three independent samples.

**Figure 1—figure supplement 3.**
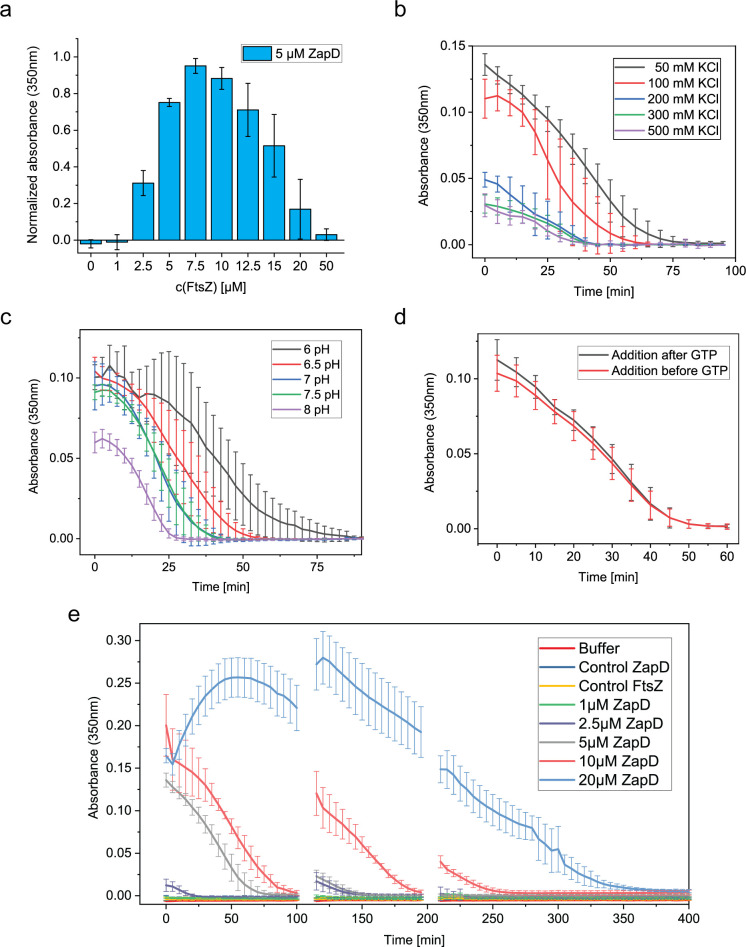
Biochemical characterization of the ZapD and FtsZ bundles by turbidity at 350 nm*.* The characterization of the FtsZ bundles resulting from the interaction with ZapD was made by measuring the turbidity of the samples at 350 nm in a plate reader. The mean value and standard deviation are the result of 3–6 independent samples. The signal from the blanks was collected before polymerization of FtsZ and subtracted for further measurements. Control of ZapD and FtsZ polymers independently did not show any significant difference with the blank after GTP addition. The working buffer was used unless it is specified in the legend (50 mM KCl, 50 mM Tris-Cl, 5 mM MgCl_2_, and pH 7). The absorbance was measured every 5 min for 100 min. (**a**) FtsZ bundling at different concentrations of FtsZ with 5 µM ZapD and 1 mM GTP in working conditions. In this case, the signal from FtsZ bundles was normalized to the maximum absorbance achieved (7.5 µM FtsZ) to facilitate the interpretation of the results. The signal plotted corresponds to 5 min after the addition of GTP. There was a clear decrease in the signal obtained at high concentration of FtsZ due to the low protein ratio ZapD/FtsZ. (**b**) Assembly of FtsZ bundles at different ionic strength conditions using KCl. The concentration of FtsZ and ZapD was 5 µM for both proteins, adding 1 mM GTP in the working buffer supplemented with different concentrations of KCl (50–500 mM KCl). Higher ionic strength in the buffer reduced the amount or size of bundles formed in solution, as the FtsZ-ZapD interaction is decreased. (**c**) Effect of pH in the FtsZ bundling process. The protein concentration used was 5 µM of ZapD and FtsZ with the addition of 1 mM GTP in the working buffer at different pH (6.5–8 pH). Lower pH enhanced the amount of FtsZ bundles promoted by ZapD, as lateral FtsZ-FtsZ interactions are promoted at lower pH. (**d**) Analysis of FtsZ bundling over time when ZapD was mixed with FtsZ prior or after the GTP addition and polymerization. The concentration of FtsZ and ZapD was 5 µM and 1 mM GTP in working buffer, respectively. ZapD mixed with FtsZ before or after GTP addition did not show significant difference in the formation of FtsZ bundles. (**e**) Measurement of turbidity over time of 5 µM FtsZ in the presence of ZapD at increasing concentrations. Both proteins were mixed prior to the addition of 1 mM GTP. A decay in the signal can be observed over time, highlighting the dynamic nature of the FtsZ bundles. After 100 min, an extra 1 mM GTP was added and FtsZ bundles were reassembled, showing lower increase of the signal due to a higher concentration of GDP in the samples, likely shifting the reaction. The addition of 1 mM GTP was done twice after 100 min.

**Figure 1—figure supplement 4.**
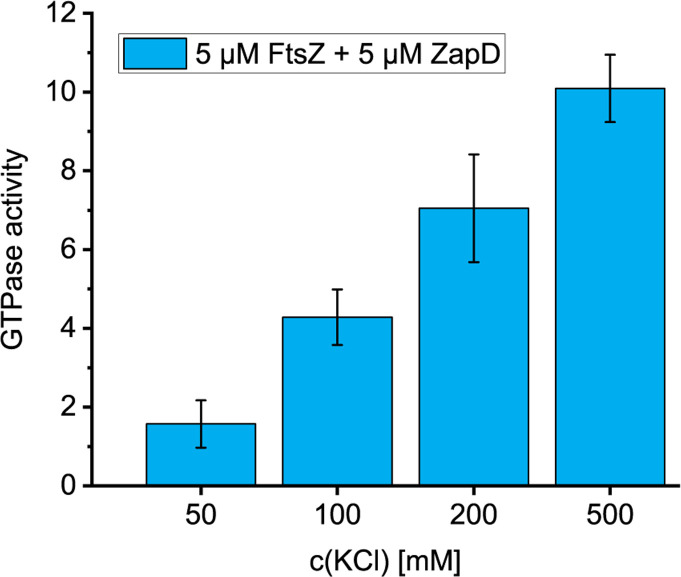
Ionic strength in the buffer lowers the effect of ZapD over FtsZ GTPase activity. GTPase activity of FtsZ in the presence of equimolar concentration of ZapD (5 µM) after the addition of 1 mM GTP at different ionic strength conditions (50–500 mM KCl, 50 mM Tris-Cl, 5 mM MgCl_2_, pH 7). The mean value and SD of the FtsZ GTPase activity correspond to 3 independent replicates. The GTPase activity was measured as a result of the Pi released from GTP consumption. The units are mol GTP consumed per mol FtsZ per minute. Moderate salt concentrations (100–200 mM) usually support optimal FtsZ assembly and GTP hydrolysis.

**Figure 1—figure supplement 5.**
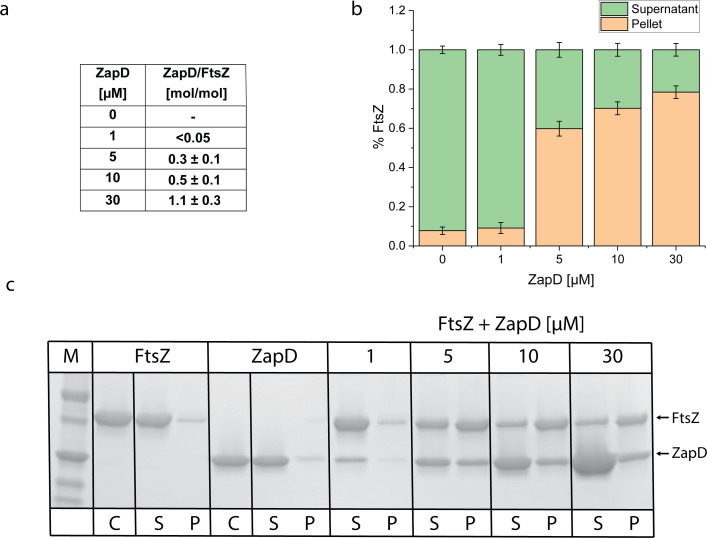
ZapD binding to FtsZ polymers via sedimentation assays. (**a**) Analytical sedimentation velocity (SV) assay. This table summarizes the binding of ZapD to FtsZ polymers, determined by the SV of FtsZ at 5 µM and increasing concentrations of ZapD (1–30 µM). The experiments were done at working conditions. (**b, c**) Pelleting assays using a preparative centrifuge. Samples of FtsZ (5 µM) and increasing concentrations of ZapD (1–30 µM) were sedimented at 10,000 rpm, and the pellets and supernatants were subjected to SDS-PAGE (see panel c). The relative intensities in the electrophoresis allowed a qualitative estimation of the concentrations of FtsZ in the supernatant and pellet. (**b**) shows the estimation of FtsZ in the pellet and supernatant. The protein concentrations and buffer conditions used in this assay were the same as those in the analytical SV assay. (**c**) The samples loaded in the SDS gel from left to right include FtsZ at 5 µM, ZapD at 10 µM, and FtsZ in the presence of 1, 5, 10, or 30 µM ZapD. The molecular weight markers from top to bottom are 50, 37, 25, 20, and 15 kDa. ‘C’ denotes the control sample without centrifugation, while ‘S’ represents the supernatant, and ‘P’ indicates the pellet. Figure 1—figure supplement 5—source data 1.Raw images of the SDS-PAGE gels used in Figure 1—figure supplement 5 and for quantification, corresponds to the raw SDS-PAGE gels. Figure 1—figure supplement 5—source data 2.Raw images of the SDS-PAGE gels used in Figure 1—figure supplement 5 and for quantification, includes the labelled images.

To date, a high-resolution structure of the Z ring remains unresolved. Most evidence suggests that FtsZ filaments are arranged in short patches along the equatorial circle, held together by lateral interactions and interactions with Zap proteins (Du and Lutkenhaus, 2019; McQuillen and Xiao, 2020; Huecas et al., 2017; Sundararajan and Goley, 2017; Szwedziak et al., 2014). The dynamics of treadmilling and lateral interactions between FtsZ filaments, as well as crosslinking by FtsZ-associated proteins (Zaps), are thought to play a crucial role in the condensation of the Z ring (Whitley et al., 2021). Specifically, the crosslinking of filaments by Zaps, particularly ZapD, is vital for maintaining the neighboring organization of the filaments into a ring structure. This organization may also be critical for the functionality of the divisome and the mechanism of force generation during cytokinesis (Levin and Janakiraman, 2021; Du and Lutkenhaus, 2019; McQuillen and Xiao, 2020; Söderström and Daley, 2017; Erickson et al., 2010; Nguyen et al., 2021; Mateos-Gil et al., 2019).

Zap proteins play overlapping roles in stabilizing the Z ring during cell division. Although they are nonessential for division, their absence leads to less compact Z rings, and the knowout of two or more Zap encoding genes simultaneously results in cell elongation (Durand-Heredia et al., 2012; Buss et al., 2013; Hale et al., 2011). Notably, these different Zap proteins do not share any sequence homology and differ in their structure and filament crosslinking mechanism (Huang et al., 2013; Schumacher et al., 2016; Schumacher et al., 2017). Additionally, Zap proteins may actively remove FtsZ from the septum during the final stages of constriction (Pazos et al., 2013). They also facilitate the positioning of the Z ring at the replication terminus of the chromosome by interacting with MatP, which forms a scaffolding anchor known as Ter-linkage (Bailey et al., 2014).

ZapD is a symmetrical dimer consisting of an α-helical domain and a β-sheet domain containing a positively charged binding pocket required for crosslinking and bundling FtsZ filaments (Roach et al., 2016; Schumacher et al., 2017). ZapD binds to the CCTP of FtsZ through electrostatic interactions (Roach et al., 2016). ZapD crosslinks FtsZ filaments, promoting bundling and significantly reducing the GTPase activity of FtsZ (Durand-Heredia et al., 2012). A model of how ZapD crosslinks neighboring FtsZ filaments has been proposed based on the structural analysis of ZapD-FtsZ interactions (Schumacher et al., 2017). In this model, the ZapD dimer connects two FtsZ filaments through the CCTP, which could organize them in a parallel or antiparallel orientation due to the flexibility provided by the flexible linker (Figure 1a). However, the experimental validation of this model is still missing.

Furthermore, the link between FtsZ bundling, promoted by ZapD, and the large-scale organization of FtsZ filaments remains unresolved. Therefore, investigating how ZapD crosslinks FtsZ filaments may shed light on the molecular mechanisms underlying Z ring assembly. Recently, Gong et al., 2024, demonstrated that although the FtsZ crosslinking proteins are not individually essential, they collectively play a critical role in the condensation and stability of the Z ring in different organisms. However, the specific mechanisms driving this condensation remain unclear, highlighting the complexity of the bacterial cell division process.

In this study, we used cryo-electron microscopy (cryo-EM) and cryo-electron tomography (cryo-ET), together with biochemical and biophysical assays, to investigate the structural organization of FtsZ polymers in the presence of ZapD. This integrated approach revealed that, at equimolar concentrations of ZapD and FtsZ, ZapD facilitates the assembly of FtsZ filaments into toroidal polymers in solution. This observation is consistent with the low-resolution structure of the Z ring obtained in vivo (McQuillen and Xiao, 2020; Fu et al., 2010; Li et al., 2007; Lyu et al., 2016). Our results allowed us to propose a molecular mechanism for toroid formation, providing valuable insights into how crosslinking of division proteins drives the stabilization of the Z ring.

## Results

### ZapD dimer interacts with FtsZ-GDP oligomers

Previous structural studies have identified a direct interaction between ZapD and FtsZ through the CCTP or central hub of FtsZ, involving charged residues in the interaction (Huang et al., 2016; Roach et al., 2016). To independently verify these findings, we investigated the interaction between FtsZ-GDP and ZapD using analytical ultracentrifugation (AUC) (see Figure 1—figure supplement 1). First, we confirmed that ZapD forms stable dimers, which is consistent with previous studies (Durand-Heredia et al., 2012; Huang et al., 2016; Schumacher et al., 2017; Figure 1—figure supplement 1aI and b). In contrast, unpolymerized FtsZ-GDP primarily self-assembles from monomers into dimers and other oligomeric species, albeit in small proportions, as expected (Rivas et al., 2000; Figure 1—figure supplement 1aII).

Sedimentation velocity analysis of mixtures of the two proteins revealed the presence of two predominant molecular species of ZapD:FtsZ complexes in solution. These complexes are compatible with ZapD dimers bound to one or two FtsZ monomers, corresponding to ZapD:FtsZ stoichiometries of 2:1 and 1:1, respectively (Figure 1—figure supplement 1aIII–IV). This observation is consistent with the proposed interaction model (Schumacher et al., 2017). Furthermore, we confirmed the interaction of both proteins using fluorescence anisotropy and fluorescence correlation spectroscopy (Figure 1—figure supplement 2a and b). In these experiments, an increase in fluorescence anisotropy or diffusion time suggested the formation of larger particles due to protein-protein interactions.

### ZapD binding promotes the bundling of FtsZ-GTP polymers

ZapD is a crosslinker for FtsZ filaments, promoting their bundling in solution (Durand-Heredia et al., 2012). However, the precise molecular mechanism by which ZapD crosslinks the filaments remains unclear. To investigate the biochemical features underlying this bundling process, we examined how the formation of large FtsZ bundles or higher-order FtsZ polymer structures depends on the concentration of ZapD. For this analysis, we used turbidity measurements at 350 nm. When testing GTP-FtsZ filaments at 5 or 10 μM concentrations, we found negligible turbidity, similar to that of ZapD alone at concentrations ranging from 1 to 40 μM. However, when ZapD was added, the turbidity of the FtsZ polymers increased, reaching a maximum at approximately 5 μM of ZapD (with a ZapD-FtsZ molar ratio of around 1). This turbidity level did not change significantly even at higher ZapD concentrations (Figure 1b, Figure 1—figure supplement 3).

In the presence of ZapD, FtsZ rapidly forms higher-order polymers upon addition of GTP, as indicated by turbidity assays. In contrast, the formation of single- or double-stranded FtsZ filaments without ZapD does not result in a significant increase in turbidity. Compared to FtsZ filaments observed in vitro, FtsZ bundles with ZapD exhibited reduced GTPase activity, reaching 20% at equimolar or higher concentrations of ZapD (Figure 1c, Figure 1—figure supplement 4), in agreement with previous studies (Durand-Heredia et al., 2012). The macrostructures formed by FtsZ in the presence of ZapD were disassembled more slowly than the FtsZ filaments upon GTP consumption (Figure 1—figure supplement 3). Replenishment of GTP allowed for a partial recovery of the turbidity signal, suggesting that the FtsZ bundles can be rapidly reassembled (Figure 1—figure supplement 3e). Previous studies have shown that high concentrations of macromolecular crowders (such as Ficoll or dextran) promote the formation of dynamic FtsZ polymer networks (González et al., 2003), observations that are consistent with our results. In these cases, the GTPase activity of FtsZ was significantly reduced compared to that of FtsZ filaments, leading to a decrease in GTPase turnover. Similar mechanisms may apply to assembly reactions involving ZapD (Durand-Heredia et al., 2012; Huang et al., 2016).

To understand the relationship between the concentration dependence of the turbidity signal – associated with the formation of FtsZ bundles or higher-order structures driven by ZapD (see Figure 1b) – and the amount of ZapD bound to the FtsZ binding sites, we carried out analytical centrifugation sedimentation velocity assays, previously used to characterize the binding of ZapD to GDP-FtsZ. This method allowed us to separate ZapD-FtsZ bundles from free ZapD in solution and calculate the concentration of unbound ZapD from the interference signal of the slowly sedimenting protein behind the rapidly sedimenting heavy ZapD-FtsZ complexes. Using the measured concentration of free ZapD together with known input concentrations of ZapD and FtsZ, we were able to calculate the concentration of bound ZapD and the binding stoichiometry of ZapD in the bundles (moles of bound ZapD in the bundles per mole of FtsZ in monomer molar units of both proteins) at different total concentrations of ZapD (see Figure 1—figure supplement 5). Our results showed that in the 5–10 μM ZapD concentration range, the bundles exhibited an estimated ZapD binding stoichiometry of approximately 0.3–0.4, meaning that one ZapD dimer is associated with every four to six FtsZ monomers. In contrast, at higher ZapD concentrations (30–40 μM), the binding stoichiometry increased to 1.1±0.2, indicating one ZapD dimer for every two FtsZ monomers. These results are qualitatively consistent with parallel co-sedimentation assays and additional analysis by SDS-PAGE, showing an increase in the relative intensity of ZapD in the pellet containing the FtsZ bundles at elevated ZapD concentrations in the mixture (see Figure 1—figure supplement 5).

These results suggest that the ZapD binding stoichiometry in the polymers increases with higher ZapD concentrations. We then carried out cryo-EM and cryo-ET studies to investigate whether this increase in binding density affects the structural organization of the bundles formed.

### ZapD facilitates the formation of higher-order FtsZ-GTP toroidal structures

**Figure 2.**
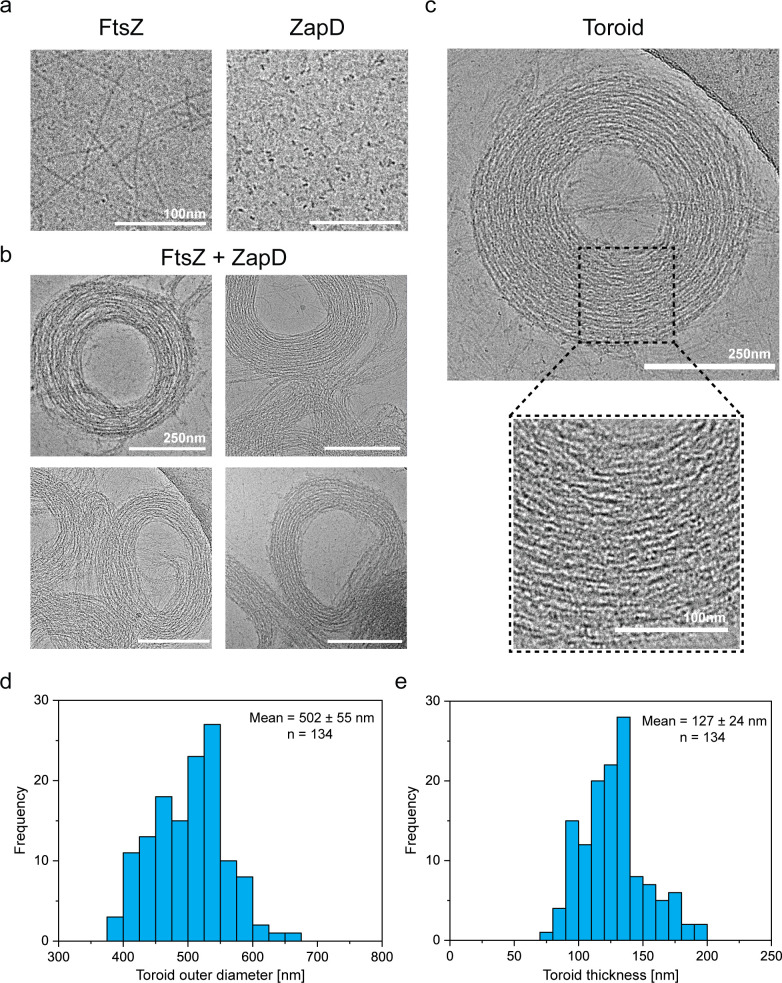
ZapD promotes the formation of FtsZ toroids. (**a**) Cryo-electron microscopy (cryo-EM) micrographs of FtsZ filaments (FtsZ-GTP form) (left) and ZapD protein (right) at 10 µM under working conditions (50 mM KCl, 50 mM Tris-Cl, 5 mM MgCl_2_, pH 7). Scale bars are 100 nm. (**b**) Cryo-EM images of FtsZ (10 µM) in the presence of equimolar concentrations of ZapD (ratio 1:1) and 1 mM GTP in working conditions. Cryo-EM grids were plunge-frozen 2 min after GTP addition to favor the assembly of FtsZ and ZapD structures. The proteins were mixed before the polymerization was triggered by GTP addition. Scale bars are 250 nm. (**c**) Micrograph of an individual FtsZ toroid found under the same conditions as in (**b**). Close-up view of an area within the toroid is framed by a dotted black line, revealing the large amount of FtsZ filaments that form its structure. (**d**) Distribution of the outer diameter of the FtsZ toroid. Each toroid was measured perpendicularly in the shortest and longest axis to ensure the reliability of the measurement. The mean value and standard deviation are shown in the graph. (**e**) Distribution of toroidal thickness. It was measured as the result of the difference between the outer and inner diameter of each toroid. The mean value and standard deviation are shown in the graph.

Previous studies have visualized bundles with similar features using negative-stain transmission electron microscopy (Durand-Heredia et al., 2012; Roach et al., 2016; Schumacher et al., 2017). Here, cryo-EM allowed us to avoid any staining artifact or structural distortion (due to adsorption or flattening upon grid drying; Kourkoutis et al., 2012) and to resolve single filaments. In the presence of GTP, FtsZ polymerized into thin filaments of variable length and curvature (Figure 2a). Upon addition of equimolar amounts of ZapD, corresponding to the slightly substoichiometric ZapD binding densities described in the previous section, FtsZ filaments assembled into bundles that predominantly formed circular toroidal structures (Figure 2b and c, Figure 2—figure supplement 1a), making them a compelling study target. A close-up view of the FtsZ toroid suggests a partially ordered arrangement of the filaments in the toroidal structure (Figure 2c).

**Figure 2—figure supplement 1.**
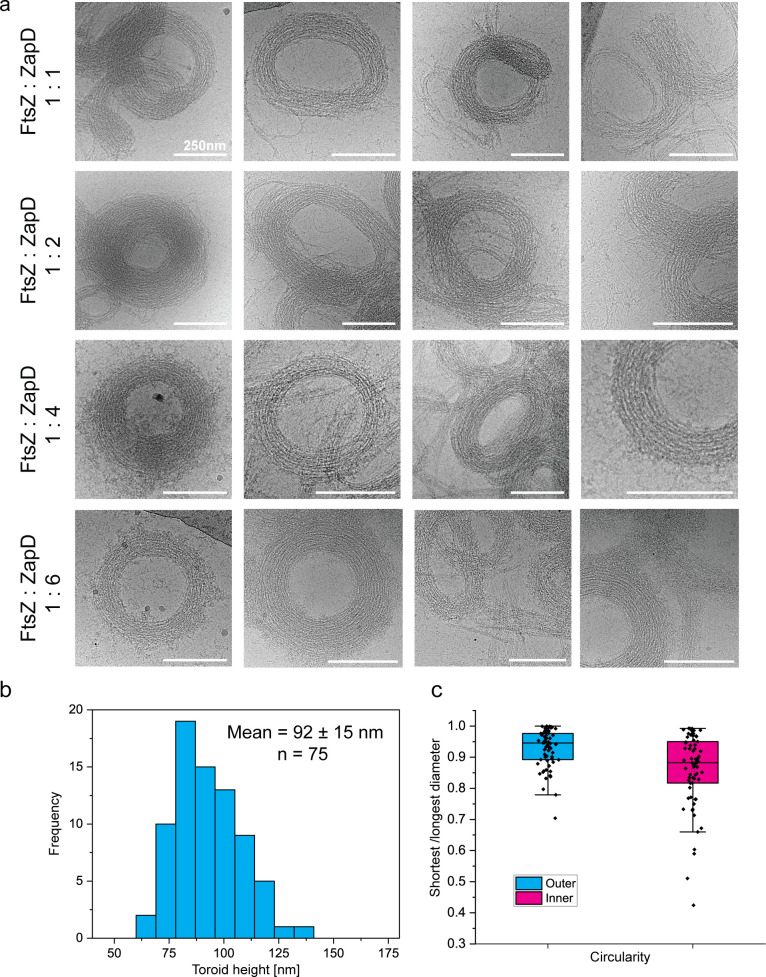
FtsZ toroids and bundles formed at different protein ratios. (**a**) Cryo-electron microscopy (cryo-EM) images of FtsZ bundles and toroids promoted by interaction with ZapD at different protein ratios. From top to bottom, the protein ratios used in the samples were 1:1, 1:2, 1:4, 1:6 for FtsZ at 10 µM and ZapD concentrations ranging from 10 to 60 µM. These ratios do not refer to the stoichiometry of the proteins forming the structure. Samples were plunge-frozen 2 min after the addition of 1 mM GTP. In these samples, similar toroidal structures and bundles were observed regardless of the protein ratio, including spirals and curved bundles. Scale bars are 250 nm. (**b**) Distribution of toroid heights measured from cryo-electron tomography (cryo-ET) data. Cross-sections of the toroids in the XZ plane were used to measure the height of each toroid. Distance measurements were taken from each toroid in the middle of the bundle at the four cardinal points to assure the reliability of the results. The mean value and SD are shown in the graph. (**c**) Circularity of FtsZ toroids. For each toroid, the shortest and largest distances of the outer and inner diameter were measured from the cryo-EM images. The division between the shortest and longest diameter provided the circularity of the FtsZ toroids. The mean value and SD are shown in the graph. The circularity of the inner and outer diameter is significantly different (t-test t(4.2), p-value<0.001).

Quantitative analysis of the toroidal structure revealed a conserved organization, with an outer diameter on the order of the bacterial cell size (502±55 nm) (Figure 2d), a typical thickness of 127±25 nm (Figure 2e), and an average height of 93±15 nm (Figure 2—figure supplement 1b). By measuring the shortest and longest axes, we determined that the circularity of the structure was 0.92±0.1 and 0.85±0.1 for the outer and inner diameters, respectively (Figure 2—figure supplement 1c). This conserved toroidal structure could result from the intrinsic curvature of the FtsZ filaments (Lu et al., 2000; Huecas et al., 2017; Erickson and Osawa, 2017) stabilized by ZapD binding. The dimension of a ZapD dimer is ~7 nm along its longest axis. Huecas et al., 2017, estimated an interfilament distance of ~6.5–6.7 nm for toroids of *Bacillus subtilis* FtsZ. These authors also observed a difference in this distance as a function of the linker, suggesting that linker length modulates FtsZ-FtsZ interactions. We observed a similar spacing for double filaments (5.9±0.8 nm) and a longer spacing in the presence of ZapD (7.88±2.1 nm). Previous studies with ZapD have suggested that distances of 6–12 nm are possible based on the protein structure (Schumacher et al., 2017). Longer linkers may also provide additional freedom to spread the filaments further apart and allow a greater degree of variability in the connections made by ZapD.

Previous studies have shown different FtsZ structures at different concentrations and buffer conditions. FtsZ filaments are flexible and can generate different curvatures ranging from mini rings of ~24 nm to intermediate circular filaments of ~300 nm or toroids of ~500 nm in diameter (reviewed in Erickson and Osawa, 2017; Wang et al., 2019). It is reasonable to assume that FtsZ filaments can accommodate the toroidal shape promoted by ZapD crosslinking.

Our data showed that ZapD dimers crosslink adjacent FtsZ filaments into bundles and toroids. These toroids are remarkably regular in size and similar to the bacterial diameter (McQuillen and Xiao, 2020; Fu et al., 2010; Li et al., 2007), suggesting a conserved intrinsic curvature of the FtsZ filaments across a range of ZapD bonds (Erickson and Osawa, 2017).

### 3D structure of ZapD-mediated FtsZ toroids revealed by cryo-ET

**Figure 3.**
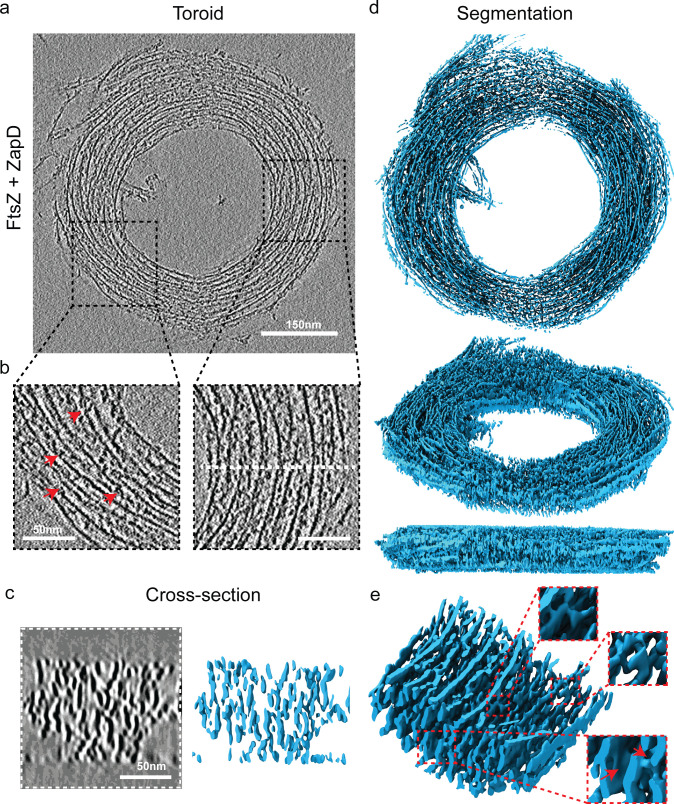
Three-dimensional (3D) structure of FtsZ toroids revealed by cryo-electron tomography (cryo-ET). (**a**) Representative tomographic slice of an FtsZ toroid resulting from the interaction of FtsZ with ZapD. The image is the average of five 0.86-nm-thick tomographic slices (total thickness of 4.31 nm) of the reconstructed tomogram around the equatorial plane of a single FtsZ toroid. The concentrations of FtsZ and ZapD were 10 µM, and 1 mM of GTP was added to initiate polymerization under working conditions (50 mM KCl, 50 mM Tris-Cl, 5 mM MgCl_2_, pH 7). (**b**) Close-up views of the toroidal structure show the alignment of the FtsZ filaments forming the toroid. Red arrows indicate the presence of connections between filaments. (**c**) The tomographic slice in the XZ plane (left) shows the cross-section corresponding to the area marked by the white dotted line in **b** This image is the average of nine tomographic slices (total thickness of 7.74 nm) from the denoised tomogram. The isosurface of the cross-section (right) shows the vertical alignment and stacking of the FtsZ filaments within the toroid. This suggests that the interaction between FtsZ filaments and ZapD is mainly along the Z direction. FtsZ filaments are represented in blue. (**d**) Isosurface of the FtsZ toroid shown in **a**. It was extracted from the reconstruction of the denoised tomographic volume and positioned in different views to facilitate its visualization: (top) front view, (middle) side view, and (bottom) lateral view. The toroid has a diameter of ~552 nm and a height of ~92 nm. (**e**) A close-up view of the segmented toroidal structure. It shows the complex internal organization of filaments assembling the toroid. It corresponds to a zone within the toroid shown in **b** on the right. Close-up views of the isosurface show different connections between filaments. The segmentation shown has a width of 136 nm × 101 nm and a height of 64 nm.

Visualization of toroidal FtsZ structures using cryo-EM revealed a complex arrangement of FtsZ filaments within the toroidal shape (Figure 2c). To gain a more detailed understanding of their three-dimensional (3D) organization, we used cryo-ET. Our initial analysis focused on ZapD-mediated toroidal FtsZ structures at equimolar concentrations of ZapD and FtsZ (Figure 3, Figure 3—figure supplement 1a).

**Figure 3—figure supplement 1.**
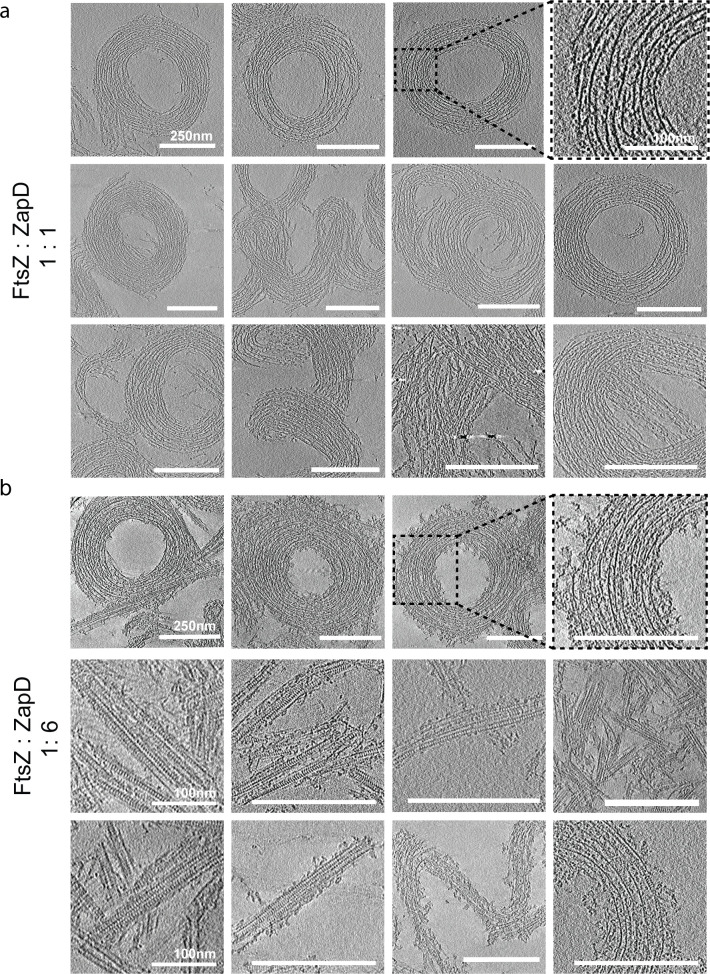
FtsZ structures found at low and high ZapD concentrations. Representative tomographic slices of FtsZ structures and bundles promoted by ZapD at different protein ratios under working conditions. The images are an average of five 0.86 nm slices from the reconstructed tomographic volume (total thickness of 4.31 nm) to enhance the signal-to-noise ratio of the images. Scale bars are 250 nm unless they are labeled with 100 nm. Samples were plunge-frozen 2 min after triggering polymerization. (**a**) Equimolar concentrations of FtsZ and ZapD (10 µm) in the sample formed toroids and bundles after 1 mM GTP addition. (**b**) High concentration of ZapD (60 µm) promoted the formation of straight FtsZ bundles, although they coexisted with scarce amounts of FtsZ toroids and double filaments showing striated patterns.

**Figure 3—figure supplement 2.**
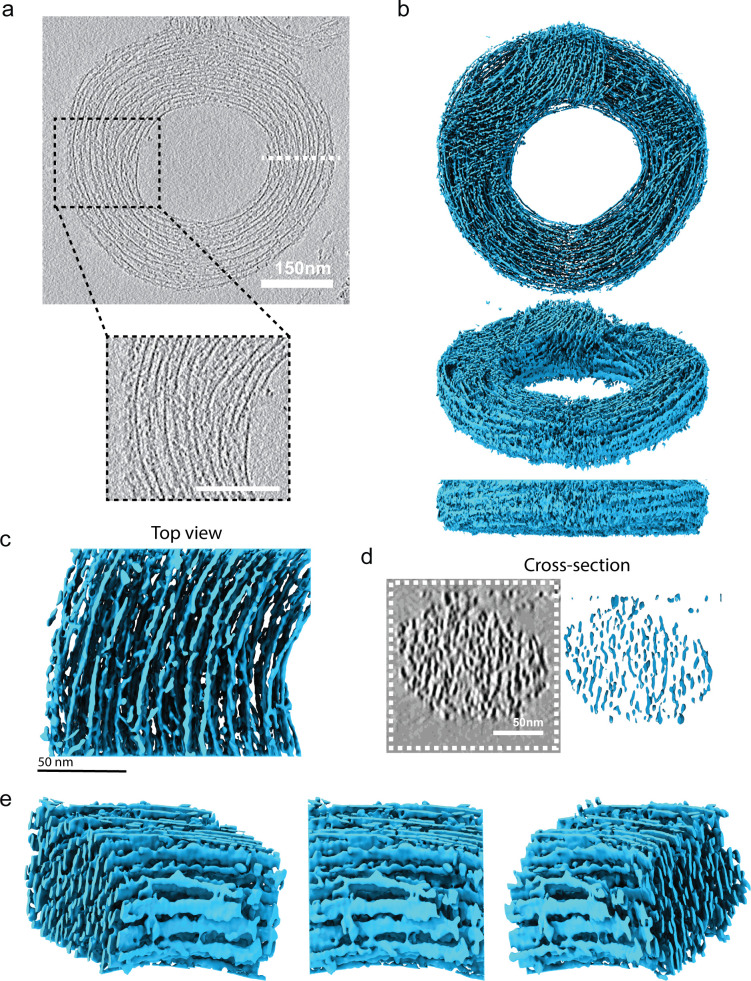
Segmentation of an FtsZ toroid imaged using cryo-electron tomography (cryo-ET). (**a**) Tomographic slice of a toroidal FtsZ structure. The image is an average of five 0.86 nm slices from the reconstructed tomographic volume (total thickness of 4.31 nm). FtsZ and ZapD were added to the sample at equimolar concentrations (10 µm) before the addition of 1 mM GTP. The white dotted line represents the localization of the cross-section shown in (**e**), while the black dotted square shows the area of a close-up view shown below the image. The scale bar of the zoomed area is 50 nm. (**b**) Corresponding segmentation of the toroid shown in **a** The isosurface of the toroid was extracted from the denoised tomographic volume and positioned in different views: front (top), side (middle), and lateral (bottom). The curved filaments observed in the upper part of the toroid correspond to a bundle that was located in the upper layer of the toroid. It has been erased to make the toroid easier to see. The toroid has a diameter of ~581 nm and a height of ~119 nm. (**c**) Segmentation of the zoomed area shown in **a**. Top view of the isosurface from the toroidal structure. (**d**) Cross-section of the toroid in the XZ plane (left; plane indicated by the white dotted line in **a**) and corresponding isosurface (right). The tomographic slice is the average of nine tomographic slices. (**e**) Different views of the isosurface of the toroidal structure. The network of filaments shown corresponds to the image shown in (**c**) in different views. The segmentation shown has a width of 116 nm × 150 nm and a height of 82 nm.

**Figure 3—figure supplement 3.**
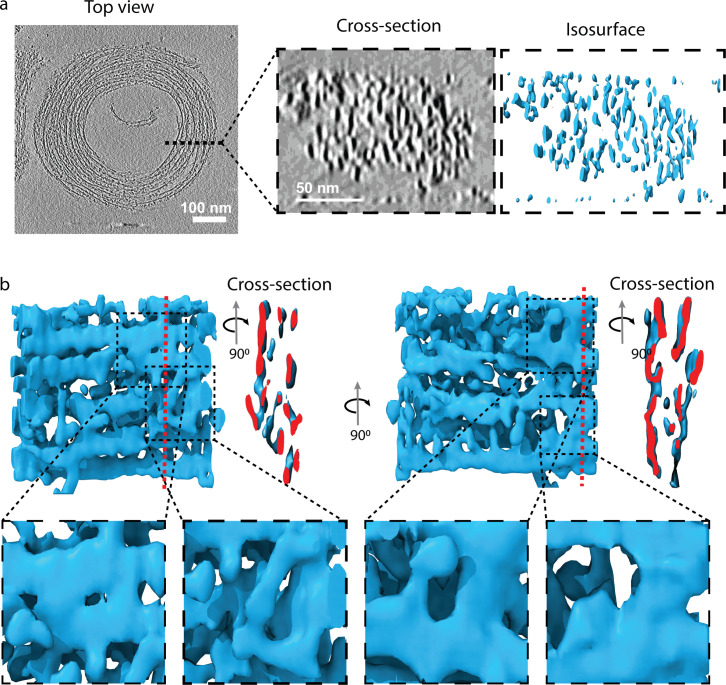
Cross-section of the toroid showing the elongated structures in the Z-axis. (**a**) Tomographic slice of a toroid. The image is an average of five 0.86 nm slices from the reconstructed tomographic volume (total thickness of 4.31 nm). The tomographic slice in the XZ plane shows a cross-section of the toroid (middle). This image is the average of nine tomographic slices (total thickness of 7.74 nm) from the denoised tomogram. Corresponding isosurface of the same area (right). (**b**) Different views of the isosurface of a filament network extracted from a toroid. The red dotted lines indicate the area that was selected for the cross-sections. Black dotted squares show closer views of the connections between filaments in the Z-plane. The segmentation shown has a width of 28 nm × 37 nm and a height of 56 nm.

**Figure 3—figure supplement 4.**
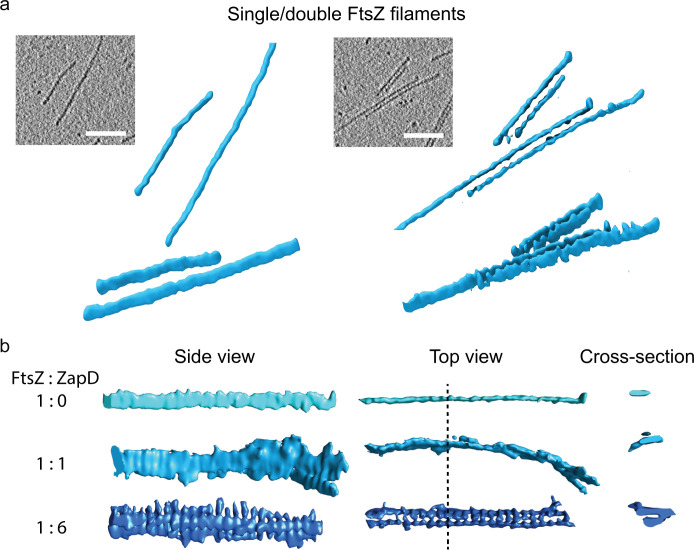
Segmentation of FtsZ filaments. (**a**) Isosurface of single and double FtsZ filaments in the absence of ZapD extracted from the denoised tomographic volume. FtsZ filaments observed from cryo-electron tomography (cryo-ET) samples in the presence of 2 mM GTP. The single and double filaments were extracted from the tomographic slice shown to the left side of each example. Top and side views of the segmented filaments are shown. Scale bars represent 50 nm. (**b**) Comparison of isolated FtsZ filaments from the following samples: (top) absence of ZapD, (middle) a toroidal structure at equimolar concentrations of FtsZ and ZapD (10 µM), and (bottom) a straight bundle at high concentration of ZapD (60 µM). The protein ratios added to the sample in each case are shown in the left side of each example. Side and top views of the three segmented filaments are shown to the left and middle columns, while a cross-section of the filaments is shown in the right column. The cross-section corresponds to the dotted line shown at the side top view. Different shades of blue distinguish the three conditions analyzed. The presence of extra densities decorating the FtsZ filaments strongly suggests that these are ZapD proteins decorating and connecting the filaments. These densities generate an elongation in Z beyond the missing wedge, which is clearly visible.

Cryo-ET revealed the dense packing of the toroidal structures, with numerous densities connecting the FtsZ filaments laterally or diagonally (Figure 3a–c, Figure 3—figure supplement 1a, and Figure 3—figure supplement 2a). Zoomed-in views of the toroids in the XY plane showed that the toroid consists of relatively short filament segments arranged nearly parallel or antiparallel to each other (Figure 3b, Figure 3—figure supplement 2a). In contrast, cross-sectional views of the toroids revealed elongated structures rather than simple filament cross-sections along the Z-axis (Figure 3c, left, Figure 3—figure supplement 3a).

We then extracted the toroid isosurface from the tomograms to visualize its 3D structure (Figure 3c, right–e, Figure 3—figure supplement 2b–e, and Video 1). The isosurface confirmed the presence of extended structures along the Z-axis, well beyond the elongation expected from the missing wedge effect for single FtsZ filaments (for comparison, see Figure 3—figure supplement 4). The vertically extended structures appeared to correspond to filaments that were connected or decorated by additional densities along the Z-axis (Figure 3—figure supplement 3b). Importantly, these densities were only observed in the presence of ZapD (Figure 3—figure supplement 4b), suggesting that they represent ZapD connections (Figure 3e, Figure 3—figure supplements 2e and 3b). We note that the resolution of the data is not sufficient to precisely resolve ZapD proteins from the FtsZ filaments in the Z-axis.

**Video 1.** Tomogram of an FtsZ toroidal structure promoted by ZapD shown in Figure 3a, followed by its segmentation. FtsZ filaments are in blue. Successive rotations of the segmented volume allow us to visualize the structure of the toroid in three dimensions (3D).

These results suggest that the toroids are constructed and stabilized by interactions between ZapD and FtsZ, which are mainly formed along the Z-axis but also vertically and diagonally.

### ZapD plasticity is essential for toroid shape and stabilization

**Figure 4.**
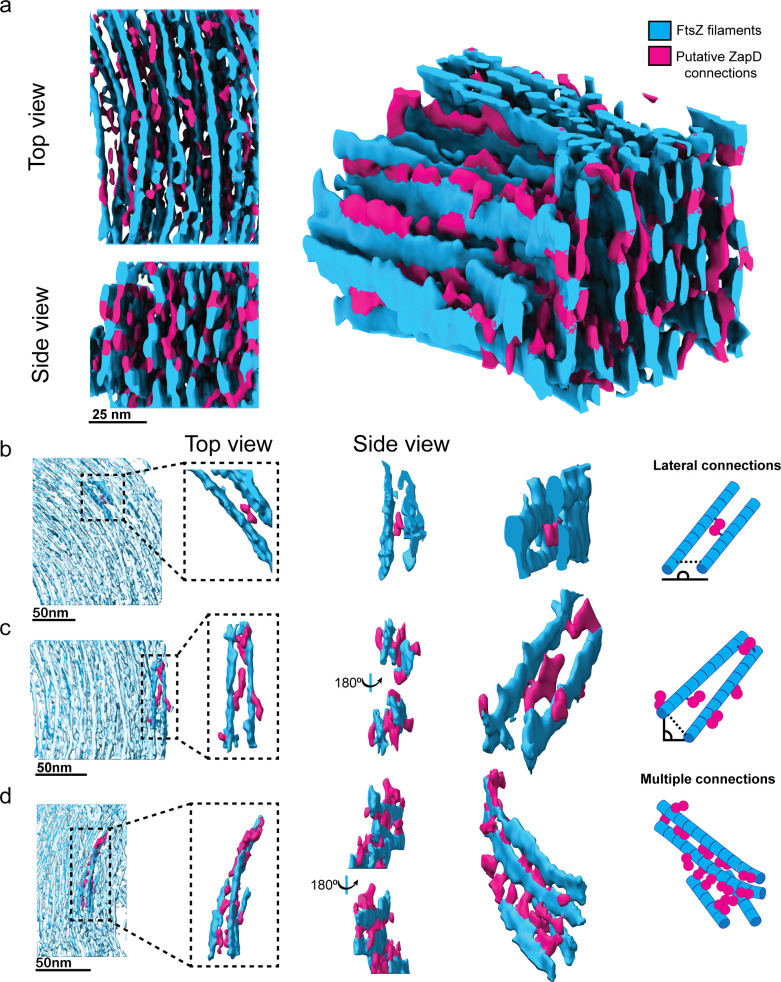
FtsZ filaments are connected by putative ZapD crosslinkers to assemble the toroidal structure. (**a**) Top (left, top), side (left, bottom), and lateral (right) views of the isosurface from a region within the toroidal structure shown in Figure 3a. The FtsZ filaments are colored in blue, while filament connections or putative ZapD proteins are labeled in magenta to facilitate interpretation of the results. Other putative ZapD proteins decorating the FtsZ filaments were not labeled in magenta because they were not forming any clear linkage between the filaments. The segmentation shown has a width of 73 nm × 101 nm and a height of 64 nm. (**b–d**) Various examples of filament connections by putative ZapD proteins within the toroid. Same color code as in (a). From left to right, the localization of the analyzed region, a close-up view of the structure of interest, different views of the crosslinkers, and a schematic illustrating the interpretation of the data. The schematic (right) shows the localization of ZapD proteins (magenta) and FtsZ filaments (blue). (**b**) Lateral connection of two FtsZ filaments by a putative ZapD dimer. In this example, the attachment of each globular density or putative ZapD monomer was bound to each filament, allowing for lateral binding. (**c**) Putative ZapD connections stabilizing two filaments by a lateral interaction. Additional ZapD decorations attached to only one of the filaments appear to be available for other filament connections. (**d**) Multiple ZapD proteins can connect to filaments and stabilize the interaction. First, the two upper filaments are connected vertically by several putative ZapDs. The lower filament connects vertically in an oblique angle to the nearest neighboring filament. In the upper part, additional decorations or putative ZapD proteins would be available to establish further interactions forming a three-dimensional (3D) mesh.

Next, we manually labeled the connecting densities in the toroid isosurfaces to analyze their arrangement and connectivity with the FtsZ filaments (Figure 4a; Video 2). The high density of the toroids and the wide variety of conformations of these densities prevented the use of subtomogram averaging to resolve their structure and spatial arrangement within the toroids. Most connections exhibited a characteristic bi-spherical shape between the filaments, reminiscent of ZapD dimers (Figure 4b–d). We observed lateral connections between two parallel FtsZ filaments at the same height within the toroid (Figure 4b). In addition, FtsZ filaments at different heights can connect vertically and diagonally through ZapD proteins acting as bridging units, forming a complex 3D mesh (Figure 4c and d and Video 2). We also identified potential ZapD proteins that decorate individual FtsZ filaments and can link them to other nearby filaments (Figure 4b). Furthermore, some filaments exhibited multiple crosslinks, leading to stronger attachments between them (Figure 4d). Estimation of the precise number of ZapD molecules per FtsZ or the number of bonds per filament is challenging due to their inherently heterogeneous organization. However, we could observe a high number of connections stabilizing the toroidal structure. The short filament length, which results in gaps between adjacent filaments, does not correlate with a higher number of ZapD connections (Figure 4—figure supplement 1), indicating that ZapD is able to crosslink filaments in all directions without causing filament breakage. This could play an important mechanistic role in the functionality of the FtsZ macrostructures.

**Figure 4—figure supplement 1.**
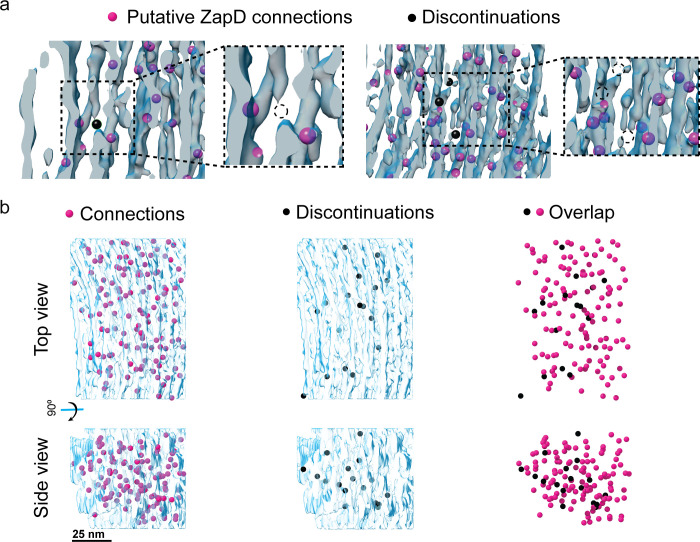
The FtsZ toroid is formed by short and discontinuous filaments crosslinked by ZapD*.* (**a**) Image of the isosurface of the network of filaments shown in Figure 4a. A black marker (sphere) is positioned at each discontinuity or termination of FtsZ filaments (blue) within the toroid. Magenta markers were placed at each clear connection between FtsZ filaments to analyze their presence in areas close to discontinuities. A closer inspection of the image, framed by a dotted black line, shows the termination and interruption of some FtsZ filaments. The black marker has been replaced by a dotted circle to show the absence of filaments in this area. The images on the left and right are just different areas of the same network of filaments within the toroid. (**b**) Images on the left and in the middle represent the spatial three-dimensional (3D) localization of discontinuities (black) and connections (magenta) of FtsZ filaments in the analyzed area of the toroid shown in (Figure 4a) (blue filaments). A top and side view of the structure shows the localization of the discontinuities and connections. The overlap of the two (right) shows no significant colocalization or correlation between them. The segmentation shown has a width of 73 nm × 101 nm and a height of 64 nm.

**Figure 4—figure supplement 2.**
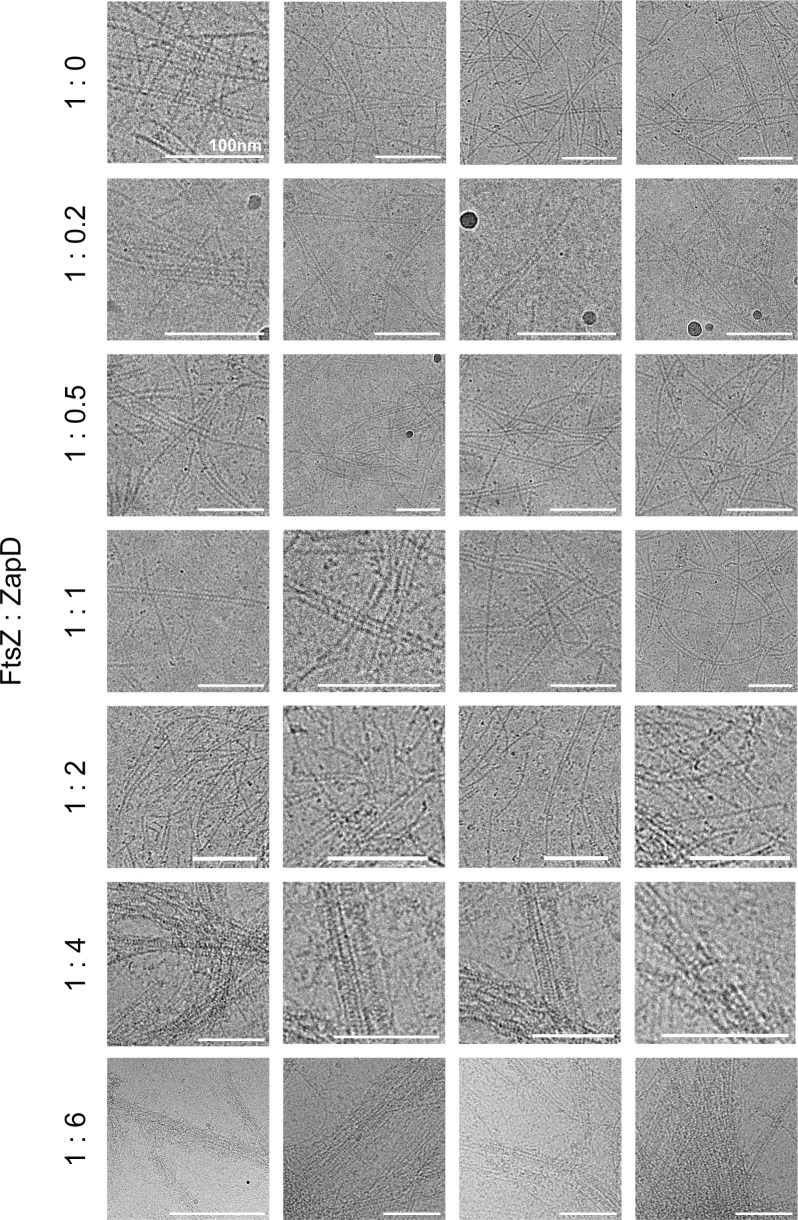
FtsZ single and double filaments found at different FtsZ and ZapD ratios. Cryo-electron microscopy (cryo-EM) images of single and double filaments found together with toroids and bundles in samples containing FtsZ and ZapD at different protein ratios in the sample. The FtsZ concentration was 10 µM, while ZapD was present at increasing concentrations (from top to bottom 0, 2.5, 5, 10, 20, 40, and 60 µM), mixed before the addition of 1 mM GTP. These ratios refer to the initial protein concentration added and not to the stoichiometry of the two proteins forming the structure. In the cryo-EM samples, only single and double filaments were found, as shown in samples from 1:0 to 1:1 protein ratio. In these filaments, we could not discard or confirm the presence of ZapD connecting the FtsZ filaments. They were not easily distinguished from double filaments formed in the absence of ZapD as a consequence of weak FtsZ-FtsZ lateral interactions. In the case of saturation of ZapD in samples containing 1:4 or 1:6, double FtsZ filaments and straight bundles with a characteristic striped pattern perpendicular to the FtsZ filaments were found. Scale bars are 100 nm.

**Video 2.** Isosurface of the toroid shown in Figure 2a. FtsZ filaments are shown in blue and putative ZapD connections in magenta. A close-up view and rotations of the segmented volume show the filament meshwork and the connections by ZapD in three dimensions (3D).

Cryo-ET imaging of ZapD-mediated FtsZ toroidal structures revealed a preferential vertical stacking and crosslinking of short FtsZ filaments, which are also crosslinked laterally and diagonally, allowing for filament curvature and resulting in a toroidal structure observed for the first time following the interaction between FtsZ and one of its natural partners in vitro.

### High concentrations of ZapD promote the structural reorganization of FtsZ polymers into straight bundles by tightly packed crosslinking

Having characterized the higher-order FtsZ structures promoted at equimolar ZapD binding densities, we addressed the effect of increasing the ZapD density on the structural organization of the ZapD-FtsZ polymers. At high concentrations of ZapD (typically 40–60 μM, representing a molar ratio of approximately one to four or six to FtsZ), we observed the formation of straight bundles with striated patterns between the FtsZ filaments (Figure 5—figure supplement 1), as well as the presence of some toroidal structures (Figure 2—figure supplement 1a). Here, the high concentration of ZapD molecules increased the number of links between the filaments and ultimately promoted the formation of straight bundles, indicating that the assembly of FtsZ-ZapD structures is a reversible process that strongly depends on the amount of ZapD proteins crosslinking the filaments. Toroids and curved bundles always coexist, but the predominance shifts from toroids to straight bundles at high ZapD concentrations. This shift suggests that the number of crosslinks between filaments can modulate the properties of the assembled higher-order structures, resulting in the reorganization of the toroidal structures into straight bundles upon ZapD saturation (Figure 4—figure supplement 2). We then explored how higher densities of ZapD lead to the formation of straight bundles using cryo-ET.

**Figure 5.**
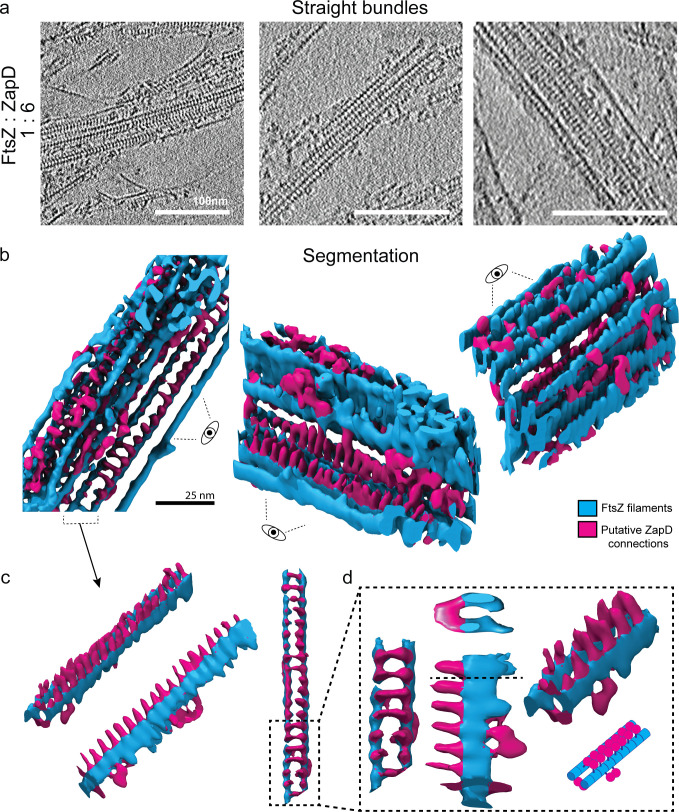
Formation of straight FtsZ bundles is driven by high ZapD crosslinking from above. (**a**) Representative tomographic slices of straight FtsZ bundles resulting from the interaction of FtsZ with high ZapD concentrations under working conditions (50 mM KCl, 50 mM Tris-Cl, 5 mM MgCl_2_, pH 7). The concentrations of FtsZ and ZapD were 10 µM and 60 µM, respectively, and 1 mM of GTP was added to trigger polymerization. The straight bundles were found only at high ZapD concentrations. The image is the average of five 0.86-nm-thick tomographic slices (total thickness of 4.31 nm) of the reconstructed tomogram. Scale bars are 100 nm. (**b**) Isosurface of the straight bundles from the denoised tomographic volume. FtsZ filaments are colored in blue and putative ZapD connections in magenta. Three different views (top [left] and side views [middle, right]) are shown. Straight bundles are organized in a regular organization. Multiple bonds between filaments are formed vertically by putative ZapDs vertically crosslinking two FtsZ filaments with a regular spacing of 4.5±0.5 nm between ZapD dimers. In addition, lateral connections were also found, connecting pairs of stabilized filaments to each other and eventually assembling the straight bundle. (**c**) Different views of one of the isolated filaments from the straight bundle. A side view of the filaments (middle) shows a spike-like structure regularly located at the top of the FtsZ filaments, connecting them vertically as observed in the top view (right). (**d**) Different close-up views of the filament structure shown in (**c**). In the cross-section of the structure (middle, top), it is clearly visible that the ZapD proteins connect the two filaments vertically and from above, forming a bridge over them. A schematic of the proposed interaction (right, bottom) shows the position of putative ZapD dimers in this structure.

Using the same approach to visualize the toroids, we collected tomograms of the straight bundles and extracted their isosurfaces (Figure 5a, Figure 3—figure supplement 1b, Figure 5—figure supplement 1, and Video 3). The straight bundles are formed at high ZapD concentrations and consist of a highly organized stack of well-aligned FtsZ filaments (Figure 5b). The connection of filaments by multiple putative ZapD proteins results in the straightening of the structure of the filaments (Lu et al., 2000; Erickson and Osawa, 2017; Figure 5c). Interestingly, ZapD crosslinks FtsZ filaments vertically, forming a row of ZapD proteins interacting with both filaments (Figure 5d). Here too, structural analysis by subtomogram averaging was not possible due to the crowding, preferential orientation, and heterogeneity of the connections. The distance between ZapD proteins provided a mean value of 4.5±0.5 nm (Figure 5—figure supplement 2a), consistent with the size of FtsZ proteins (Huecas et al., 2007). This observation suggests that most FtsZ proteins interact with the ZapD dimers that crosslink the filaments, in agreement with the ZapD enrichment in the bundles observed by sedimentation assays (Figure 1—figure supplement 5). In addition, we found that the presence of ZapD increased the distance between two FtsZ filaments connected by ZapD proteins compared with the spacing in the absence of ZapD, regardless of the amount of ZapD connections (from 5.9±0.8 to 7.88±2 nm in toroids and 7.6±1.5 nm in straight bundles; Figure 5—figure supplement 2b). This indicates that ZapD not only connects neighboring FtsZ filaments but spreads them apart, which could have important implications for the functionality of the Z ring.

**Figure 5—figure supplement 1.**
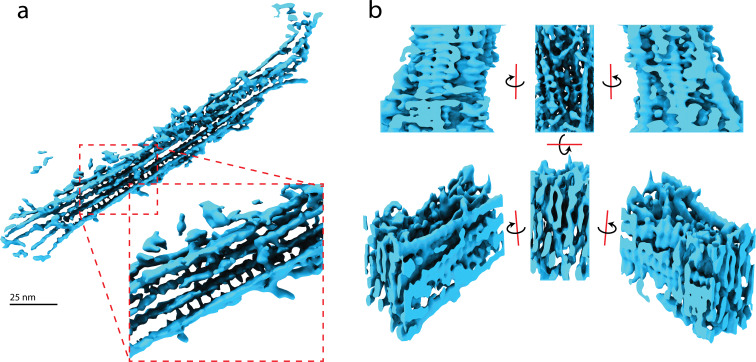
Segmentation of straight bundles imaged by cryo-electron tomography (cryo-ET). Segmentation of straight bundles resulting from the interaction of FtsZ with high concentrations of ZapD under working conditions (50 mM KCl, 50 mM Tris-Cl, 5 mM MgCl_2_, pH 7). The concentrations of FtsZ and ZapD were 10 and 60 µM, respectively. 1 mM of GTP was added to induce polymerization and bundle formation. (**a, b**) show the isosurface of two straight bundles from the denoised tomographic volume. The entire volume has been colored in blue without differentiating ZapD connections to facilitate the interpretation. (**b**) shows different views of a cropped area within a straight bundle. The segmentation shown in (**a**) has a width of 270 nm × 175 nm and a height of 72 nm. Segmentation in (**b**) has a width of 36 nm × 65 nm and 60 nm in height.

**Figure 5—figure supplement 2.**
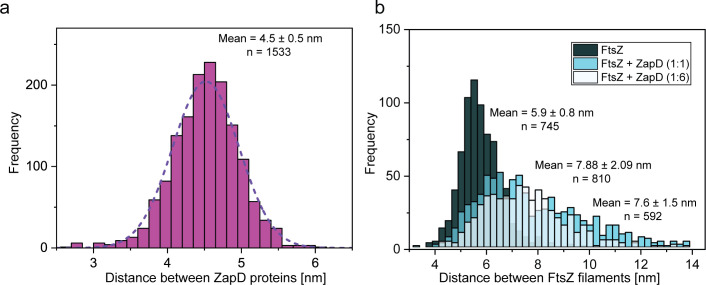
Distance between FtsZ filaments and ZapD-associated filaments*.* (**a**) Distribution of the distance between two ZapD proteins in a straight bundle under ZapD saturation (1:6 protein ratio). The distance between two ZapD proteins was measured from tomographic images obtained in samples containing FtsZ (10 µM) in the presence of ZapD at high concentration (60 µM) in the presence of GTP. The distribution obtained fitted a normal distribution. The mean value and SD are shown in the graph. The regular distance found between the ZapD proteins connecting the filaments could indicate the presence of one ZapD dimer per FtsZ monomer forming the filament. (**b**) Distribution of the distance between two FtsZ filaments (5.9±0.8 nm) in the absence or presence of ZapD forming toroidal structures (1:1) (7.88±2.09 nm) or straight bundles (1:6) (7.6±1.5 nm). The distance between filaments was measured from the cryo-electron microscopy (cryo-EM) and cryo-electron tomography (cryo-ET) images and plotted in the graph as a distribution of distances. The three distributions were obtained from images of >3 independent samples. The mean value and SD are shown in the graph for each condition. The presence of ZapD crosslinking filaments increases the distance between them compared to weak FtsZ-FtsZ interactions. However, no significant differences were found between equimolar and saturation levels of ZapD.

**Figure 5—figure supplement 3.**
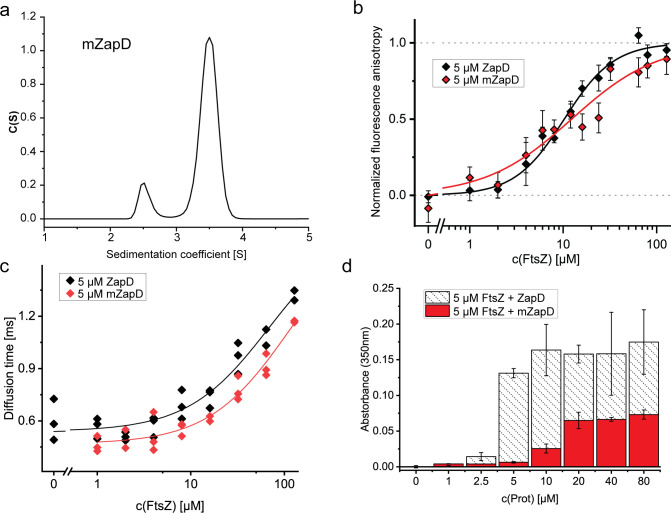
mZapD binds FtsZ-GDP and can promote bundling of FtsZ filaments. (**a**) Sedimentation coefficient distribution, *c*(*s*), obtained by sedimentation velocity (SV) with mZapD at different concentrations under working conditions (50 mM KCl, 50 mM Tris-Cl, 5 mM MgCl_2_, pH 7). mZapD sedimented mostly as a single species with an experimental sedimentation coefficient of 3.5 S when corrected to standard conditions (s20, w: 3.6 S±0.1). However, species at around 2.5 S appear at ~10%, in contrast to ZapD, demonstrating the presence of monomers and confirming the weakening of the dimerization interface. (**b**) Molecular interaction between FtsZ-GDP and ZapD or ZapD mutant (mZapD) measured by fluorescence anisotropy and compared with the results shown in Figure 1—figure supplement 2a for ZapD and FtsZ-GDP. 5 µM of mZapD was supplemented with 150 nM of mZapD-ATTO 647 N and FtsZ in increasing concentrations. mZapD (red) also binds FtsZ-GDP with an apparent *Kd* of 12.42±5.45 (SE). The X-axis is expressed in log units. The mean value and standard deviation are the result of 3 independent samples. (**c**) Fluorescence correlation spectroscopy (FCS) analysis of increasing concentrations of FtsZ-GDP with 5 µM of ZapD or mZapD supplemented with 100 nM ZapD or mZapD chemically labeled with ATTO 647 N. The plot corresponding to ZapD was the same as shown in Figure 1—figure supplement 2b and it is included here to facilitate the interpretation of the results. The X-axis is expressed in log units. The diffusion curve showed an increasing diffusion time of mZapD with increasing FtsZ concentration, supporting a direct interaction of this protein with FtsZ-GDP. The plotted line is to guide the eye only. Each condition was measured in three independent samples. (**d**) Turbidity signal of FtsZ and ZapD by measuring absorbance at 350 nm after 5 min of GTP addition. The concentration used was 5 µM FtsZ with increasing concentrations of ZapD (0–80 µM) and 1 mM GTP in working buffer. Data represents the mean and standard deviation of >3 independent samples. The signal shown for ZapD is the same as that shown in Figure 1b and it is included here to aid interpretation.

**Figure 5—figure supplement 4.**
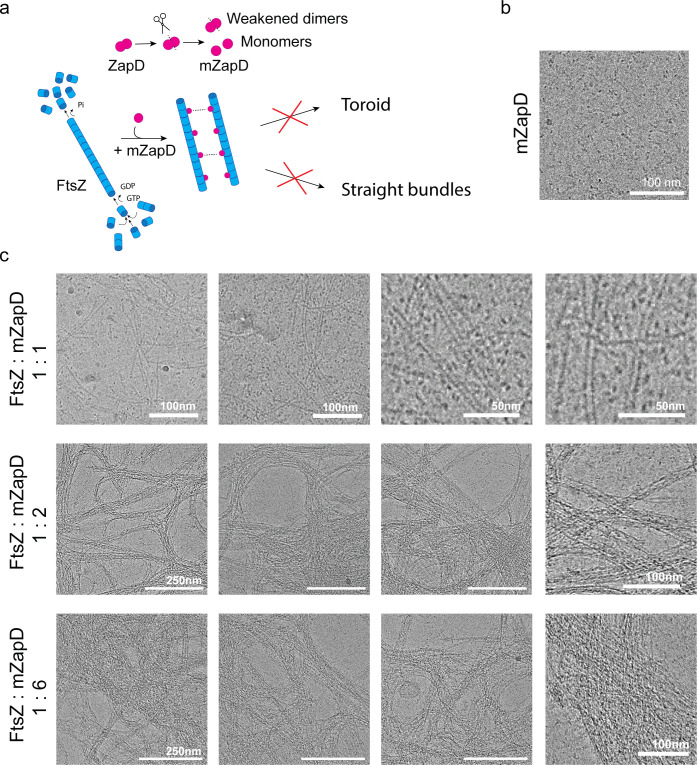
mZapD can bundle FtsZ without promoting toroids. (**a**) Proposed schematic of the interaction mechanism between FtsZ-GTP and the mZapD protein. The mZapD protein is mutated at the dimerization site to weaken dimerization and favor monomerization of the protein. Thus, the interaction of dynamic FtsZ filaments with mZapD monomers is weakened, and the stabilization of toroidal structures or straight bundles is not possible, while dynamic bundles can still be formed. (**b**) Cryo-electron microscopy (cryo-EM) image of 10 µM mZapD. mZapD is homogeneously distributed, and it did not aggregate under our working conditions. The scale bar is 100 nm. (**c**) Representative cryo-EM images of FtsZ and mZapD at increasing concentrations of mZapD and 1 mM GTP under working conditions. The ratios shown on the left side of the images refer to the initial added protein concentration. At equimolar concentrations of FtsZ and ZapD (10 µM), no FtsZ bundles were found, and only single and double FtsZ filaments were observed (top). At higher concentrations of mZapD (20 and 60 µM, middle and bottom rows, respectively), FtsZ bundles were formed, although no toroids or straight bundles were observed in these samples. The absence of these structures suggested that a stable dimer is required to form them. Scale bars are 250 nm unless 100 or 50 nm labels are shown.

**Video 3.** Tomogram of an FtsZ straight bundle formed at a high concentration of ZapD proteins shown in Figure 5b. Successive segmentation of the tomogram with FtsZ filaments labeled in blue and putative ZapD connections in magenta. Rotations and close-up views help the interpretation of the data and show the three-dimensional (3D) structure of the straight bundle.

**Figure 6.**
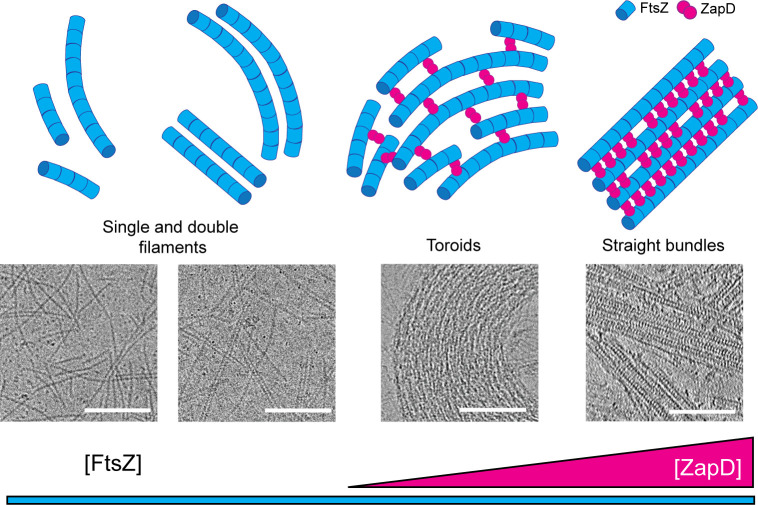
The amount of ZapD connections modulates the spatial organization of FtsZ filaments into higher-order structures. (Top) Simplified scheme of the higher-order FtsZ structures formed in the presence of increasing concentrations of ZapD. These schemes do not consider all the possible interactions between ZapD and FtsZ. (Bottom) Cryo-electron microscopy (cryo-EM) images of the structures shown in the schemes. In the absence of ZapD, FtsZ filaments can interact laterally to form double filaments upon GTP binding. At low concentrations of ZapD, only a few ZapD-mediated bonds are formed, favoring the formation of small, curved bundles. At equimolar concentrations of ZapD and FtsZ, more ZapD-mediated bonds are formed, particularly from above the filaments, but also laterally and diagonally, allowing filament curvature and favoring the assembly of toroidal structures. At saturating ZapD concentrations, the filaments are straightened up by regular ZapD crosslinking vertically, resulting in the formation of straight bundles. Overall, the assembly of higher-order FtsZ structures depends on the number of vertical crosslinks through ZapD dimers. Some intermediate states are expected between the structures shown. Scale bars are 100 nm.

These results point out the relevant role of the number of connections between filaments (driven by ZapD binding) in the crosslinking mechanism (Figure 6). The bundles formed at the high ZapD concentrations tested (up to four molar excess of ZapD to FtsZ) correspond to a binding saturation regime in which a ZapD dimer binds two FtsZ molecules connecting two filaments, straightening their structure and arranging them into large, highly organized straight bundles. In contrast, fewer connections provide the freedom to form toroidal structures. This observation confirms that a certain number of ZapD-driven bonds are optimal for maintaining the preferential curvature of FtsZ filaments and allowing toroid assembly, establishing a structural dependence of assembled bundles on the number of ZapD-mediated bonds.

### Dimerization of ZapD is essential for the formation of organized higher-order FtsZ structures

Our results suggest that ZapD dimers connect two FtsZ filaments, although the role of dimerization has not yet been experimentally established (Schumacher et al., 2017). We therefore investigated whether the dimerization of ZapD was essential for assembling straight bundles and toroidal structures. To this end, we produced a ZapD mutant (‘mZapD’) by substituting three amino acids (R20, R116, and H140) involved in its dimerization by alanine, thus decreasing the stability of the dimerization interface. The dimerization of mZapD was tested by AUC analysis, which showed a percentage of monomeric protein that was not present in the wild-type ZapD (Figure 5—figure supplement 3a). mZapD can interact with unpolymerized FtsZ-GDP as evidenced by fluorescence anisotropy and FCS (Figure 5—figure supplement 3b and c). mZapD is still able to promote FtsZ bundle formation, showing an ~50% lower turbidity signal than for wild-type ZapD even at higher concentrations (Figure 5—figure supplement 3d). We also visualized these bundles by cryo-EM and observed the formation of thinner bundles (Figure 5—figure supplement 4b and c). However, toroidal structures and straight bundles were absent in these samples even at high concentrations of mZapD. This indicates that a stable dimerization interface is required to assemble complex ZapD-mediated structures (Figure 5—figure supplement 4a).

These results demonstrated that dimerization of ZapD is essential to promote the assembly of organized high-order FtsZ structures such as toroids and straight bundles, highlighting the structural importance of stable ZapD-driven connections between filaments.

## Discussion

In this study, we used biochemical reconstitution, cryo-EM, and cryo-ET to gain new insights into the structure and assembly of the bacterial divisome. Filament crosslinking by Zap proteins plays a crucial role in the assembly and stabilization of the Z ring. However, the underlying mechanisms of this process remain to be elucidated. To better understand these mechanisms, we investigated the interaction between FtsZ and ZapD, one of the stabilizers of the division ring.

Our results indicate that ZapD crosslinks FtsZ in solution to form 3D toroidal structures characterized by short, discontinuous FtsZ filaments that are vertically stacked and crosslinked, as well as diagonally and laterally crosslinked, as revealed by cryo-ET. The relative concentrations of FtsZ and ZapD dictate the assembly geometry. Remarkably, FtsZ toroids emerge as the dominant structure when both proteins are present in equimolar ratios, whereas straight bundles are assembled at high concentrations of ZapD. Our study highlights the importance of the balance between vertical and lateral or diagonal crosslinking to allow for the filament curvature observed in the toroids, which is absent in the straight bundles where vertical crosslinking is dominant.

The structural characterization of the toroids by cryo-ET allowed us to resolve the overall structure and identify putative ZapD dimers that crosslink the filaments. However, we were unable to obtain detailed structural information about the ZapD connectors due to the heterogeneity and density of the toroidal structures, which showed significant variability in the conformations of the connections between the filaments in all directions. These results are consistent with the observation that ZapD interacts with FtsZ through its central hub, which provides additional spatial freedom to connect other filaments in different conformations. This flexibility allows different filament organizations and contributes to structural heterogeneity. In addition, these results suggest that these crosslinkers can act as modulators of the dynamics of the ring structure, spacing filaments apart and allowing them to slide in an organized manner (Szwedziak et al., 2014; Nguyen et al., 2021; Lan et al., 2009; Hörger et al., 2008). The ability of FtsZ to treadmill directionally (Whitley et al., 2021; Loose and Mitchison, 2014; Ramirez-Diaz et al., 2018; Yang et al., 2017; Bisson-Filho et al., 2017), together with the parallel or antiparallel arrangement of short, transiently crosslinked filaments, is considered essential for the functionality of the Z ring and its ability to exert constrictive force (Whitley et al., 2021; Erickson et al., 2010; Nguyen et al., 2021; Mateos-Gil et al., 2019; Erickson and Osawa, 2017). Thus, Zap proteins can play a critical role in ensuring correct filament placement and stabilization, which is consistent with the toroidal structure formed by ZapD.

Our observation of toroidal FtsZ structures promoted by ZapD in solution is consistent with the observation of pre-curved FtsZ protofilaments in circular assemblies attached to model membranes in various in vitro systems (Loose and Mitchison, 2014; Ramirez-Diaz et al., 2018; Vélez, 2022). Additionally, FtsZ can form ring-shaped structures with diameters ranging from 100 to 200 nm on surfaces, becoming more pronounced when adsorbed to lipid, carbon, or mica surfaces (Huecas et al., 2007; Mingorance et al., 2005; González et al., 2005), or in the presence of molecular crowders like methylcellulose (Popp et al., 2009). Cryo-EM of concentrated (50 μM) FtsZ from *B. subtilis* with GMPCPP revealed that these protofilaments often coalesce into spirals or toroids, forming large aggregates (Huecas et al., 2017). These toroidal structures correspond to the curved conformations of FtsZ polymers observed in various bacterial species, which are thought to contribute to the generation of constriction forces (Erickson and Osawa, 2017). In *E. coli,* approximately 5000 FtsZ monomers (about one third of the intracellular concentration of FtsZ) could circle the cell six to eight times (Stricker et al., 2002), suggesting a discontinuous toroidal assembly. ZapD, present at approximately 500 molecules per cell (Durand-Heredia et al., 2012), represents only a small fraction of the FtsZ molecules and favors the formation of toroidal structures in the absence of interactions with other FtsZ-associated proteins. Higher concentrations of ZapD would be required to form straight bundles.

The persistence length and curvature of FtsZ filaments are optimized to form bacterial-sized ring structures (McQuillen and Xiao, 2020; Erickson and Osawa, 2017). ZapD helps to stabilize these toroids by crosslinking and increasing the spacing between FtsZ filaments from 5.9±0.8 to 7.9±2 nm in toroids and 7.6±1.5 nm in straight bundles (Figure 5—figure supplement 2b). This increased spacing promotes a more dynamic organization, providing functional flexibility in bacteria. The linker connecting ZapD to FtsZ can also modulate filament spacing and curvature (Huecas et al., 2017; Sundararajan and Goley, 2017; Sundararajan et al., 2018). We observe a mixture of curvatures in the internal organization of the toroids. While FtsZ filaments have a preferred curvature, the density of ZapD connections forces the structure to adapt and align with neighboring filaments, thereby affecting FtsZ curvature. However, the precise molecular mechanism linking ZapD binding and polymer curvature remains to be elucidated.

One of the most significant findings of this study is that the amount of ZapD bound to FtsZ filaments greatly influences the structural organization of the resulting higher-order polymer. At lower concentrations of ZapD, which are necessary for bundling (equimolar mixtures of ZapD and FtsZ), toroids are the most prominent structures, with a binding stoichiometry of ZapD in the polymer of 0.3–0.4 moles of ZapD per mole of FtsZ (equivalent to one ZapD dimer for every four to six FtsZ molecules). As the concentration of ZapD increases, the binding stoichiometry of ZapD in the polymers increases to 1.1 moles of ZapD per mole of FtsZ (one ZapD dimer for every two FtsZ molecules). This leads to a reorganization of the polymer bundles, resulting in straight structures that predominantly crosslink the FtsZ filaments, a configuration not seen at lower ZapD concentrations (Figure 5). The increase in the number of ZapD-FtsZ contacts likely reduces the flexibility needed to form toroidal structures, compelling the filaments to adopt a larger, straight bundle conformation. We observed slight differences in the spacing between filaments (Figure 5—figure supplement 2b), with a broader range of distances between filaments in the toroids compared to the straight bundles. Thus, ZapD contributes to the variability of spacing based on the number of connections formed.

We therefore hypothesize that the assembly of functional, curved FtsZ macrostructures occurs only within a specific stoichiometric range of ZapD-FtsZ interactions. An increase in crosslinking at higher ZapD concentrations appears to cause the FtsZ polymer to form rigid, straight bundles (Figure 6). Additionally, only a robust ZapD dimer can form FtsZ toroids and straight filaments, indicating that a certain binding strength is necessary to bundle the filaments and maintain their structure. These observations, along with the high conformational variability found in ZapD connections, suggest that ZapD can modulate the behavior of the entire structure through a concentration-dependent mechanism. This structural reorganization highlights the polymorphic nature of the assemblies formed by FtsZ (Mateos-Gil et al., 2019; Monterroso et al., 2013).

We also demonstrated that ZapD-mediated FtsZ toroids replicate some of the structural features of the Z ring architecture observed in vivo. Based on super-resolution imaging and cryo-ET, the current model of the Z ring describes a somewhat discontinuous and heterogeneous structure composed of randomly overlapping filaments (Szwedziak et al., 2014; Fu et al., 2010; Rowlett and Margolin, 2014). These filaments arrange into a belt-like macromolecular entity, containing nodes with a higher density of dispersed filaments confined to a toroidal zone approximately 80–100 nm wide (toroidal height) and about 40–60 nm thick, located 13–16 nm below the inner membrane (Szwedziak et al., 2014; Buss et al., 2013; Fu et al., 2010; Lyu et al., 2016; Rowlett and Margolin, 2014; Holden et al., 2014; Szwedziak and Ghosal, 2017). The toroidal structures promoted by ZapD exhibit dimensions within this range. However, their thickness and multilayering are significantly greater, likely due to the absence of other cellular components competing for binding with FtsZ. The Z ring model also suggests that FtsZ filaments are weakly associated with one another through protein factors such as Zaps and weak FtsZ-FtsZ interactions, which may be crucial for their functionality (McQuillen and Xiao, 2020; Huecas et al., 2017; Sundararajan and Goley, 2017; Haeusser et al., 2015). Our findings indicate that one of the Zap proteins can stabilize and enhance associations between neighboring FtsZ filaments. Studying macrostructures stabilized by natural crosslinkers is vital for understanding the molecular function of the division machinery.

We believe that the intrinsic features of the toroidal structures share commonalities with the bacterial division ring. Despite the differences and limitations that may arise from an in vitro approach, the structures observed following ZapD crosslinking of FtsZ filaments may reveal inherent features that occur in vivo. The current model of the division ring consists of an array of filaments loosely connected by crosslinkers at the center of the cell, forming a ring. This model is consistent with our findings, although many questions remain regarding the structural organization of the Z ring within the cell. ZapD binds to FtsZ vertically, allowing either ZapD or FtsZ to interact with the plasma membrane. In straight bundles, this facilitates the stacking of straight FtsZ filaments, while for toroids, ZapD can also bind FtsZ filaments diagonally. This less compact arrangement could allow bending of the FtsZ filaments and adjustment of the toroid size.

The binding of ZapA, a key member of the FtsZ-associated proteins family, to FtsZ in equimolar mixtures induces the effective alignment and straightening of FtsZ filaments tethered to lipid bilayers by FtsA protein, as demonstrated through high-resolution fluorescence microscopy and precise quantitative image analysis (Caldas et al., 2019). This interaction reveals a structure comprising one ZapA tetramer for every four FtsZ molecules in the polymer, highlighting the organized nature of these interactions. It is noteworthy that these straight, parallel polymers – manifested when ZapA occupies all binding sites – bear striking resemblance to the straight bundles identified in this study at high concentrations of ZapD, indicative of binding saturation. Understanding how the associative states of ZapA (as tetramers) and ZapD (as dimers), together with membrane tethering, influence the predominant structures formed in both systems is essential. The complexity of the division system raises important questions about the interaction dynamics between FtsZ and the plasma membrane. The competitive nature of the division components to engage with FtsZ and modulate its functionality remains to be thoroughly elucidated. It is important to note that FtsA and ZipA have a greater affinity for the CCTP of FtsZ than ZapD. Our cryo-ET data on straight bundles provide new perspectives on how ZapD-FtsZ structures can effectively bind to the plasma membrane; in particular, the CCTP of parallel FtsZ filaments is oriented upward, allowing direct membrane binding or interaction with ZapDs that reinforce these filaments from above, rather than from the side, as previously suggested (Schumacher et al., 2017).

In conclusion, this study extends the understanding of the intricate structural organization and filament crosslinking of FtsZ polymers facilitated by ZapD, which is critical for the formation and maintenance of the division ring. The revelation that ring morphology depends on the binding stoichiometry between FtsZ and the crosslinking ZapD is invaluable, particularly as we integrate this knowledge into current reconstitution assays in membrane systems. This knowledge is central to unraveling the role of FtsZ-associated proteins in coordinating and maintaining division ring formation. In addition, our findings could greatly enhance the understanding of how polymeric cytoskeletal networks are remodeled during essential cellular processes such as cell motility and morphogenesis. Although conventional wisdom points to molecular motors as the primary drivers of filament remodeling through energy consumption, there is increasing evidence that there are alternative mechanisms that do not rely on such energy, instead harnessing entropic forces via diffusible crosslinkers (Braun et al., 2016). This approach may also be applicable to ZapD and FtsZ polymers, suggesting a promising avenue for optimizing conditions in the reverse engineering of the division ring to enhance force generation in minimally reconstituted systems aimed at achieving autonomous cell division.

## Methods

### Protein purification

ZapD protein has been overproduced and purified following the procedure previously described in Durand-Heredia et al., 2012, with some modifications. The bacterial strain was an *E. coli* BL21 (DE3) transformed with pET28b-h10-smt3-ZapD fusion plasmid (Durand-Heredia et al., 2012). The cells were sonicated for 6–8 cycles of 20 s and centrifuged at 40,000 rpm in MLA 80 rotor (Beckman Coulter) for 45 min. The protein was eluted by chromatography in 5 mL HisTrap (GE Healthcare) and digested with Smt3-specific protease Ulp1 (SUMO protease encoded in Rosetta pTB145) at 1:1 molar relation. Digestion proceeded for two dialysis sessions of 1 hr at 4°C to avoid protein precipitation. ZapD and His-Smt3 were separated by chromatography in 5 mL HisTrap (GE Healthcare), then the protein was eluted in a 5 mL HiTrap Desalting column (Healthcare) to eliminate completely possible traces of free phosphates in the buffer. Final concentration of ZapD was determined by absorbance at 280 nm using an extinction coefficient (*ε*) of 25230 M^–1^ cm^–1^, and ZapD purity was checked by SDS-PAGE. Protein fractions were frozen in buffer 50 mM KCl, 50 mM Tris-Cl, 10 mM MgCl_2_, 0.2 mM DTT, and 2% glycerol. ZapD mutant (mZapD) was purified following the same procedure using the corresponding plasmid. *E. coli* FtsZ was overproduced and purified following the calcium precipitation method described previously (Rivas et al., 2000). Briefly, *E. coli* BL21 (DE3) pLysS cells were transformed with pET11b-FtsZ, grown in LB medium, and selected with Ampicillin 100 μg/mL. After induction and growth, the pellet was resuspended in PEM buffer (50 mM PIPES-NaOH, pH 6.5, 5 mM MgCl_2_, 1 mM EDTA) and disrupted using a tip sonicator for 3–4 cycles. The lysate was then separated by centrifugation for 30 min at 20,000 × *g* at 4°C, and the supernatant was mixed with 1 mM GTP, 20 mM CaCl_2_, and incubated at 30°C for 15 min to induce FtsZ polymerization and bundling. Subsequently, the FtsZ bundles were pelleted by centrifugation for 15 min at 20,000 × *g* at 4°C, and the pellet was resuspended in PEM buffer and centrifuged again for 15 min at 20,000 × *g*, 4°C, collecting the supernatant. Precipitation and resuspension steps were repeated to improve the purification. The buffer was then exchanged using an Amicon Ultra-0.5 centrifugal filter unit 50 kDa (Merck KGaA). FtsZ purity was checked by SDS-PAGE, and concentration was determined by absorbance at 280 nm using an extinction coefficient (*ε*) of 14000 M^–1^ cm^–1^ (Rivas et al., 2000). Protein solutions were aliquoted, frozen in liquid nitrogen, and stored at –80°C until further use.

### Plasmid design and molecular cloning

To generate the ZapD mutant (mZapD), seamless cloning method was used according to the provider’s protocol (Thermo Fisher Scientific/Invitrogen GeneArt Seamless Cloning and Assembly Enzyme Mix [A14606]) using the plasmid pET28b-h10-smt3-ZapD and the following primers (R20A) ‘GAAAAAATGCGTACATGGCTGGCTATTGAGTTTT’, ‘GGCAATTTCTGCGTGAAGAT**GCT**TTGATTGCTC’, and ‘CTTTGATTTACCTACATTG**GCT**ATTTGGCTGC’ to substitute the amino acids 20 (R), 116 (R), and 240 (H) for A. All enzymes for cloning were from Thermo Fisher Scientific (Waltham, MA, USA). Briefly, DNA fragments were amplified with Phusion High-Fidelity DNA Polymerase and origo primers (Sigma-Aldrich, St. Louis, MO, USA). Then, PCR products were treated with DpnI and combined using GeneArt Seamless Cloning and Assembly Enzyme Mix. The plasmid was propagated in *E. coli* OneShot TOP10 (Thermo Fisher Scientific) and purified using NucleoBond Xtra Midi kit (Macherey-Nagel GmbH, Düren, Germany). Directed site mutagenesis was made by substituting three amino acids in the ZapD sequence by Alanine (R20, R116, H140). The plasmid was then verified using Sanger Sequencing Service (Microsynth AG, Balgach, Switzerland).

### Protein labeling

The protein was covalently labeled at the amine groups with Alexa Fluor 488 carboxylic acid succinimidyl ester, a fluorescent dye (Molecular Probes/Invitrogen), under conditions that ensured minimal interference of the dye with FtsZ assembly, as described in Reija et al., 2011. ZapD and mZapD were labeled with ATTO-647N carboxylic acid succinimidyl ester dye in the amino group. Before the reaction, ZapD was dialyzed in 20 mM HEPES, 50 mM KCl, 5 mM MgCl_2_, pH 7.5, and the probe was dissolved in dimethylsulfoxide. The reaction was allowed to proceed for 35–60 min at room temperature (RT) and stopped with 10% Tris-HCl 1 M. The free dye was separated from labeled protein by a Hi-TRAP Desalting column (GE Healthcare). The final degree of labeling of FtsZ and ZapD was calculated from the molar absorption coefficients of the protein and the dye. It was around 0.5 moles of probe per mole of FtsZ and around 0.3/0.4 moles of dye per mole of ZapD.

### AUC, sedimentation velocity, and SE

Sedimentation velocity assays were performed to detect the homogeneity and association state of individual proteins and the stoichiometry of the formed protein-protein complexes. In brief, the experiments were carried out at 43–48 K rpm in an Optima XL-I analytical ultracentrifuge, equipped with UV-Vis absorbance and Rayleigh interference detection systems. The sedimentation coefficient distributions were calculated by least-squares boundary modeling of sedimentation velocity data using the *c*(*s*) method (Schuck, 2000) as implemented in the SEDFIT program. The s-values of the present species were corrected to standard conditions (pure water at 20°C, and extrapolated to infinite dilution) to obtain the corresponding standard s-values (s20,w) using the program SEDNTERP (Laue et al., 1992). Multi-signal sedimentation velocity data were globally analyzed by SEDPHAT software (Schuck, 2003) using the ‘multi-wavelength discrete/continuous distribution analysis’ model, to determine the spectral and diffusion deconvoluted sedimentation coefficient distributions, *ck*(*s*), from which the stoichiometry of protein complexes can be derived (Balbo et al., 2005). Sedimentation equilibrium (SE) of ZapD was carried out to confirm the association state of the protein in the same experimental conditions and concentration range tested by sedimentation velocity (2–30 µM). Short columns (100 µL) SE experiments were carried out at 14,000 and 10,000 rpm. Weight-average buoyant molecular weights were obtained by fitting a single-species model to the experimental data using the HeteroAnalysis program (Cole, 2004), once corrected for temperature and buffer composition with the program SEDNTERP (Laue et al., 1992).

### Turbidity assay

Turbidity of protein samples was collected by measuring the absorbance at 350 nm in a TECAN plate reader (Tecan Group Ltd., Mannedorf, Switzerland). All samples reached a final volume of 50 μL in a 364-Well Flat-Bottom Microplate (UV-Star, Greiner Bio-One GmbH) before measuring the absorbance. Different concentrations of FtsZ and ZapD were premixed in the well plate and measured before addition of GTP to extract subsequently the individual blank values. Concentrations of FtsZ and ZapD varied from 0 to 80 μM, and buffer conditions are specified in each graph and the caption of the figures ranging from 50 to 500 mM KCl, 6–8 pH, always supplemented with 50 mM Tris-Cl and 5 mM MgCl_2_. Manual mixing was performed after addition of GTP, and orbital oscillations for 5 s in the TECAN were made prior to any measurement to avoid sedimentation of the samples. FtsZ and ZapD alone do not generate significant differences with the blank. Time measurements were taken as specified in each condition. Reported values are the average of 3–12 independent measurements ± standard deviation.

### GTPase activity of FtsZ

GTPase activity of FtsZ was measured by quantification of the inorganic phosphate with a colorimetric assay (BIOMOL GREENÒ kit from ENZO Life Sciences) for 2 min. 5 μM FtsZ was used in our standard buffer (5 mM MgCl_2_, 50 mM Tris-HCl, 50 mM KCl, pH 7) or buffers at higher KCl concentrations (50–500 mM KCl) and polymerization was triggered by 1 mM GTP. ZapD was added at different concentrations and premixed with FtsZ before addition of GTP. 13 μL fractions were added to a 96-Well Flat-Bottom Microplate (UV-Star, Greiner Bio-One GmbH) every 20 s after addition of GTP and mixed with the Reaction buffer reaching 50 and 100 μL of BIOMOL GREEN reagent, to stop the reaction. After stopping the reaction, samples were incubated for 10 min at RT, and the absorbance was measured at 620 nm in a Varioskan Flash plate reader (Thermo Fisher Scientific, MA, USA). Concentrations of inorganic phosphate were calculated from a phosphate standard curve, while the GTPase activity reaction rate (V, mol P/mol FtsZ/min) was determined from the slope of the linear part of phosphate accumulation curves.

### FtsZ sedimentation assay

Purified ZapD (1–30 μM) was added to purified FtsZ (5 μM) in the working buffer (50 mM KCl, 50 mM Tris-HCl, 5 mM MgCl_2_), and GDP or GTP (1 mM) was added last to trigger FtsZ polymerization. The reaction mixtures with a final volume of 100 μL were processed at RT and centrifuged at low speed (10,000 rcf) using a tabletop centrifuge. At that point, 90 μL of supernatant was carefully collected and loaded in clean tubes with 1x loading dye. The rest of the supernatant was discarded, and the pellets were resuspended in the original reaction volume buffer plus 1× loading dye (final concentration). The supernatants (10 μL) and pellets (10 μL) were resolved in a SDS-PAGE gel. The amount of FtsZ was estimated by image analysis using ImageJ.

### Preparation of EM grids

Cryo-EM grids were plunge-frozen with a Vitrobot Mk.IV (Thermo Fisher Scientific) using 3 µL of the samples applied to previously glow-discharged R 2/1 Holey Carbon Cu 200 mesh EM grids (Quantifoil). Samples were 10 µM FtsZ with or without ZapD or mZapD at different concentrations specified in each case (0–60 µM). Proteins were mixed in our working buffer containing 50 mM, 5 mM MgCl_2_, 50 mM Tris-HCl, pH 7. Samples were incubated for 2 min after the addition of 1 mM GTP to trigger polymerization. The Vitrobot was set to 4°C, 100% humidity, blot time 3 s, blot force 3. Whatman no. 1 filter paper was used for blotting, and liquid ethane kept at liquid nitrogen temperatures was used as a cryogen for vitrification.

### Cryo-EM and cryo-ET

Cryo-EM/ET data were acquired on two microscopes as follows. Cryo-EM micrographs were acquired within SerialEM (Mastronarde, 2005) on a Talos Arctica transmission electron microscope (Thermo Fisher Scientific) operated at 200 kV, equipped with a Falcon III (Thermo Fisher Scientific) direct electron detector operated in integrating mode. Images were recorded at ×73,000 magnification (pixel size 1.997 Å) and ×92,000 magnification (pixel size 1.612 Å) at –2.5 to –5 µm target defocus with an approximate total electron dose of 60 electrons/Å².

Cryo-EM micrographs and cryo-ET tilt series were acquired with SerialEM on a Titan Krios G2 transmission electron microscope (Thermo Fisher Scientific) operated at 300 kV, equipped with a FEG, post-column energy filter (Gatan), and a K3 camera (Gatan) operated in electron counting mode. Micrographs were recorded at ×42,000 magnification (pixel size 2.154 Å) at –5 µm target defocus with an approximate total electron dose of 60 electrons/Å². Tilt series were recorded at ×42,000 magnification (pixel size 2.154 Å) at –5 µm target defocus with an approximate total electron dose of 100–140 electrons/Å². Acquisition was performed using a dose-symmetric tilt scheme, a tilt range of ±60°, an angular increment of 2°.

### Tomogram reconstruction

Tilt series preprocessing was performed using the TOMOMAN package (https://github.com/wan-lab-vanderbilt/TOMOMAN, Wan, 2024). In brief, MotionCor2 (Zheng et al., 2017) was used to align individual frames, followed by cumulative dose weighting using an exposure-dependent attenuation function, as described in Schur et al., 2016. Dose-weighted tilt series were aligned using IMOD (Kremer et al., 1996) either by employing gold fiducials (if available) or by patch tracking, and binned tomograms (pixel size 8.616 Å) were generated using weighted back projection. Stacks were split into odd and even frames to generate half-set tomograms which were used for training and subsequent denoising in cryoCARE (Buchholz et al., 2019).

Denoised tomograms were used directly for segmentation of the toroids, straight bundles, and individual filaments by cropping an area of interest and displaying the volume as an isosurface in UCSF ChimeraX (Goddard et al., 2018). Thresholds for volume extraction were 80–100 and the color used was #00AEEF (light blue) for FtsZ and #EC008C (magenta) for ZapD isosurfaces. Small particles were removed by using the ‘dust’ function with a size of 10. Different perspectives and zooms of the structures were manually adjusted for each figure in ChimeraX. To highlight connections between filaments, the corresponding parts with putative ZapD connections were manually cropped from the volume using the Volume Eraser tool in UCSF Chimera (Pettersen et al., 2004). Both cropped and original isosurfaces were superimposed to show the colored putative ZapDs in the structure. The missing wedge effect induces an elongation by a factor of 2 along the Z-axis. This elongation is observed in the filaments of the 1:0 ratio toroids, whereas the elongation observed in the filaments of the 1:1 and 1:6 ratio toroids exceeds the missing wedge.

All micrographs and slices through tomograms were visualized using IMOD. Isosurface renderings of toroids, straight bundles, and individual filaments were displayed using UCSF ChimeraX and UCSF Chimera.

### Fluorescence anisotropy

Anisotropy measurements were performed using a TECAN plate reader (Tecan Group Ltd., Mannedorf, Switzerland). Excitation and emission wavelengths were 625 and 680 nm, respectively. ZapD or mZapD labeled with ATTO 647 N were used as fluorescence tracer with a final concentration of 150 nM of ATTO-647N and supplemented with unlabeled ZapD reaching a concentration of 5 μM. FtsZ was added at increasing concentrations to analyze their interaction. Binding affinities (apparent *K*_*d*_) were determined by fitting the Hill equation to the normalized anisotropy data. Each condition was measured in three independent samples.

### Fluorescence correlation spectroscopy

FCS measurements were performed using a PicoQuant MicroTime200 system equipped with an Olympus 60x, NA1.2 UPlanApo water immersion objective. A pulsed 636 nm diode laser was used to excite fluorescence of Atto647N-labeled ZapD, mZapD, or free Atto647N carboxylate (for calibration). Three measurements of 60 s each were performed per sample at RT (21°C), and three samples per condition were measured. The repetition rate of the pulsed laser was 26.7 MHz, and the average power was 1 µW at the objective back pupil. Fluorescence was collected through the same objective, spatially filtered through a 50 µm diameter pinhole, and spectrally filtered through a 690/70 nm bandpass filter before being split by a neutral beam splitter onto two avalanche photodiodes (Excelitas SPCM-AQRH-14-TR). Photon count events were digitized using a PicoQuant TimeHarp 260 Nano TCSPC card. Time-correlated single photon counting information was used for monitoring data quality during measurements, but not in further analysis. Data was subjected to a burst removal algorithm similar to Margineanu et al., 2007, and only ‘non-burst’ data was used in correlation analysis to obtain statistics representative of the large number of small oligomer particles, ignoring rare large ones. Cross-correlation functions of the two detectors were calculated and fitted with a standard model for 3D diffusion in with rapid dye blinking:

$$\displaystyle G\left (\tau \right)=G_{0}\left [\frac{1- F_{B}+F_{B}e^{\frac{- \tau }{\tau _{B}}}}{1- F_{B}}\right ]\frac{1}{1+\frac{\tau }{\tau _{d}}}\sqrt{\frac{1}{1+\frac{\tau }{S^{2}\tau _{d}}}}$$

With amplitude $G_{0}$, diffusion time $\tau _{d}$, point spread function aspect ratio $S$, and blinking parameters $F_{B}$ and $\tau _{B}$. Custom software was written in Python for burst removal, correlation, and fitting, based on tttrlib 0.0.19 (https://github.com/Fluorescence-Tools/tttrlib, Peulen and Hemmen, 2024). The software is in ongoing development and available upon request.

### Image analysis

Electron microscopy images were processed and analyzed using IMOD and ImageJ. The dimensions of the toroidal structures, FtsZ bundles, and distances between filaments were manually measured using ImageJ and IMOD. Distances were plotted as histograms using Origin (OriginPro, Version 2019b. OriginLab Corporation, Northampton, MA, USA). For each toroid analyzed (*n*=67), the inner and outer diameters were measured by collecting the major and minor distances in each case. The circularity of the toroid was the result of the division between the minor diameter divided by major diameter for each toroid. The height of the FtsZ toroid was manually measured from the tomograms, collecting four measurements per toroid to assure a correct representation of the size (*n*=17). For the spacing between filaments, the space in between the filaments was measured manually in the XY plane by using IMOD. Each measurement represents only one FtsZ double filament or one FtsZ-ZapD double filament; however, the same bundle could be measured more than once as they are composed of multiple filaments. The measurements were collected from >3 independent samples. The distances between ZapDs connecting two FtsZ filaments were measured following the same methodology. The mean value and standard deviation of different datasets were calculated and added to the figures together with the *n* used for each case.

## Data Availability

The data sets for all experimental conditions and graphs generated in this study are shown in the manuscript or provided in the supplementary materials/source data file.

## Additional files

MDAR checklist

Source data 1. Raw data used to make the graphs shown in figures.

## References

[bib1] Addinall SG, Lutkenhaus J (1996). FtsA is localized to the septum in an FtsZ-dependent manner. Journal of Bacteriology.

[bib2] Attaibi M, den Blaauwen T (2022). An updated model of the divisome: regulation of the septal peptidoglycan synthesis machinery by the divisome. International Journal of Molecular Sciences.

[bib3] Bailey MW, Bisicchia P, Warren BT, Sherratt DJ, Männik J (2014). Evidence for divisome localization mechanisms independent of the Min system and SlmA in *Escherichia coli*. PLOS Genetics.

[bib4] Balbo A, Minor KH, Velikovsky CA, Mariuzza RA, Peterson CB, Schuck P (2005). Studying multiprotein complexes by multisignal sedimentation velocity analytical ultracentrifugation. PNAS.

[bib5] Bisson-Filho AW, Hsu Y-P, Squyres GR, Kuru E, Wu F, Jukes C, Sun Y, Dekker C, Holden S, VanNieuwenhze MS, Brun YV, Garner EC (2017). Treadmilling by FtsZ filaments drives peptidoglycan synthesis and bacterial cell division. Science.

[bib6] Braun M, Lansky Z, Hilitski F, Dogic Z, Diez S (2016). Entropic forces drive contraction of cytoskeletal networks. BioEssays.

[bib7] Buchholz TO, Jordan M, Pigino G, Jug F (2019). Cryo-CARE: content-aware image restoration for cryo-transmission electron microscopy data.

[bib8] Buske PJ, Levin PA (2012). Extreme C terminus of bacterial cytoskeletal protein FtsZ plays fundamental role in assembly independent of modulatory proteins. The Journal of Biological Chemistry.

[bib9] Buss J, Coltharp C, Huang T, Pohlmeyer C, Wang S-C, Hatem C, Xiao J (2013). In vivo organization of the FtsZ-ring by ZapA and ZapB revealed by quantitative super-resolution microscopy. Molecular Microbiology.

[bib10] Caldas P, López-Pelegrín M, Pearce DJG, Budanur NB, Brugués J, Loose M (2019). Cooperative ordering of treadmilling filaments in cytoskeletal networks of FtsZ and its crosslinker ZapA. Nature Communications.

[bib11] Choi H, Min K, Mikami B, Yoon HJ, Lee HH (2016). Structural and biochemical studies reveal a putative FtsZ recognition site on the Z-ring Stabilizer ZapD. Molecules and Cells.

[bib12] Cole JL (2004). Analysis of heterogeneous interactions. Methods in Enzymology.

[bib13] de Boer PA, Crossley RE, Rothfield LI (1989). A division inhibitor and a topological specificity factor coded for by the minicell locus determine proper placement of the division septum in *E. coli*. Cell.

[bib14] de Boer P, Crossley R, Rothfield L (1992). The essential bacterial cell-division protein FtsZ is a GTPase. Nature.

[bib15] Du S, Lutkenhaus J (2019). At the heart of bacterial cytokinesis: the Z ring. Trends in Microbiology.

[bib16] Durand-Heredia JM, Yu HH, De Carlo S, Lesser CF, Janakiraman A (2011). Identification and characterization of ZapC, a stabilizer of the FtsZ ring in *Escherichia coli*. Journal of Bacteriology.

[bib17] Durand-Heredia J, Rivkin E, Fan G, Morales J, Janakiraman A (2012). Identification of ZapD as a cell division factor that promotes the assembly of FtsZ in *Escherichia coli*. Journal of Bacteriology.

[bib18] Ebersbach G, Galli E, Møller-Jensen J, Löwe J, Gerdes K (2008). Novel coiled-coil cell division factor ZapB stimulates Z ring assembly and cell division. Molecular Microbiology.

[bib19] Erickson HP, Anderson DE, Osawa M (2010). FtsZ in bacterial cytokinesis: cytoskeleton and force generator all in one. Microbiology and Molecular Biology Reviews.

[bib20] Erickson HP, Osawa M (2017). FtsZ constriction force - curved protofilaments bending membranes. Sub-Cellular Biochemistry.

[bib21] Fu G, Huang T, Buss J, Coltharp C, Hensel Z, Xiao J (2010). In vivo structure of the *E. coli* FtsZ-ring revealed by photoactivated localization microscopy (PALM). PLOS ONE.

[bib22] Goddard TD, Huang CC, Meng EC, Pettersen EF, Couch GS, Morris JH, Ferrin TE (2018). UCSF ChimeraX: Meeting modern challenges in visualization and analysis. Protein Science.

[bib23] Gong H, Yan D, Cui Y, Li Y, Yang J, Yang W, Zhan R, Wan Q, Wang X, He H, Chen X, Lutkenhaus J, Yang X, Du S (2024). The divisome is a self-enhancing machine in *Escherichia coli* and Caulobacter crescentus. Nature Communications.

[bib24] González JM, Jiménez M, Vélez M, Mingorance J, Andreu JM, Vicente M, Rivas G (2003). Essential cell division protein FtsZ assembles into one monomer-thick ribbons under conditions resembling the crowded intracellular environment. The Journal of Biological Chemistry.

[bib25] González JM, Vélez M, Jiménez M, Alfonso C, Schuck P, Mingorance J, Vicente M, Minton AP, Rivas G (2005). Cooperative behavior of *Escherichia coli* cell-division protein FtsZ assembly involves the preferential cyclization of long single-stranded fibrils. PNAS.

[bib26] Haeusser DP, Rowlett VW, Margolin W (2015). A mutation in *Escherichia coli* ftsZ bypasses the requirement for the essential division gene zipA and confers resistance to FtsZ assembly inhibitors by stabilizing protofilament bundling. Molecular Microbiology.

[bib27] Hale CA, Boer P (2002). ZipA is required for recruitment of FtsK, FtsQ, FtsL, and FtsN to the septal ring in *Escherichia coli*. Journal of Bacteriology.

[bib28] Hale CA, Shiomi D, Liu B, Bernhardt TG, Margolin W, Niki H, de Boer PAJ (2011). Identification of *Escherichia coli* ZapC (YcbW) as a component of the division apparatus that binds and bundles FtsZ polymers. Journal of Bacteriology.

[bib29] Holden SJ, Pengo T, Meibom KL, Fernandez Fernandez C, Collier J, Manley S (2014). High throughput 3D super-resolution microscopy reveals Caulobacter crescentus in vivo Z-ring organization. PNAS.

[bib30] Hörger I, Velasco E, Rivas G, Vélez M, Tarazona P (2008). FtsZ bacterial cytoskeletal polymers on curved surfaces: the importance of lateral interactions. Biophysical Journal.

[bib31] Huang KHH, Durand-Heredia J, Janakiraman A (2013). FtsZ ring stability: of bundles, tubules, crosslinks, and curves. Journal of Bacteriology.

[bib32] Huang KH, Mychack A, Tchorzewski L, Janakiraman A (2016). Characterization of the FtsZ C-terminal variable (CTV) region in Z-ring assembly and interaction with the Z-ring stabilizer ZapD in *E. coli* cytokinesis. PLOS ONE.

[bib33] Huecas S, Schaffner-Barbero C, García W, Yébenes H, Palacios JM, Díaz JF, Menéndez M, Andreu JM (2007). The interactions of cell division protein FtsZ with guanine nucleotides. The Journal of Biological Chemistry.

[bib34] Huecas S, Ramírez-Aportela E, Vergoñós A, Núñez-Ramírez R, Llorca O, Díaz JF, Juan-Rodríguez D, Oliva MA, Castellen P, Andreu JM (2017). Self-Organization of FtsZ polymers in solution reveals spacer role of the disordered C-Terminal Tail. Biophysical Journal.

[bib35] Kourkoutis LF, Plitzko JM, Baumeister W (2012). Electron microscopy of biological materials at the nanometer scale. Annual Review of Materials Research.

[bib36] Kremer JR, Mastronarde DN, McIntosh JR (1996). Computer visualization of three-dimensional image data using IMOD. Journal of Structural Biology.

[bib37] Lan G, Daniels BR, Dobrowsky TM, Wirtz D, Sun SX (2009). Condensation of FtsZ filaments can drive bacterial cell division. PNAS.

[bib38] Laue TM, Shah BD, Ridgeway TM, Pelletier SL (1992). Analytical Ultracentrifugation in Biochemistry and Polymer Science.

[bib39] Levin PA, Janakiraman AL (2021). Localization, assembly, and activation of the *Escherichia coli* Cell division machinery. EcoSal Plus.

[bib40] Li Z, Trimble MJ, Brun YV, Jensen GJ (2007). The structure of FtsZ filaments in vivo suggests a force-generating role in cell division. The EMBO Journal.

[bib41] Loose M, Mitchison TJ (2014). The bacterial cell division proteins FtsA and FtsZ self-organize into dynamic cytoskeletal patterns. Nature Cell Biology.

[bib42] Löwe J, Amos LA (1999). Tubulin-like protofilaments in Ca2+-induced FtsZ sheets. The EMBO Journal.

[bib43] Lu C, Reedy M, Erickson HP (2000). Straight and curved conformations of FtsZ are regulated by GTP hydrolysis. Journal of Bacteriology.

[bib44] Lyu Z, Coltharp C, Yang X, Xiao J (2016). Influence of FtsZ GTPase activity and concentration on nanoscale Z-ring structure in vivo revealed by three-dimensional Superresolution imaging. Biopolymers.

[bib45] Ma X, Margolin W (1999). Genetic and functional analyses of the conserved C-terminal core domain of *Escherichia coli* FtsZ. Journal of Bacteriology.

[bib46] Margineanu A, De Feyter S, Melnikov S, Marchand D, van Aerschot A, Herdewijn P, Habuchi S, De Schryver FC, Hofkens J (2007). Complexation of lipofectamine and cholesterol-modified DNA sequences studied by single-molecule fluorescence techniques. Biomacromolecules.

[bib47] Mastronarde DN (2005). Automated electron microscope tomography using robust prediction of specimen movements. Journal of Structural Biology.

[bib48] Mateos-Gil P, Tarazona P, Vélez M (2019). Bacterial cell division: modeling FtsZ assembly and force generation from single filament experimental data. FEMS Microbiology Reviews.

[bib49] McQuillen R, Xiao J (2020). Insights into the structure, function, and dynamics of the bacterial cytokinetic FtsZ-Ring. Annual Review of Biophysics.

[bib50] Mingorance J, Tadros M, Vicente M, González JM, Rivas G, Vélez M (2005). Visualization of single *Escherichia coli* FtsZ filament dynamics with atomic force microscopy. The Journal of Biological Chemistry.

[bib51] Monterroso B, Alfonso C, Zorrilla S, Rivas G (2013). Combined analytical ultracentrifugation, light scattering and fluorescence spectroscopy studies on the functional associations of the bacterial division FtsZ protein. Methods.

[bib52] Mukherjee A, Lutkenhaus J (1999). Analysis of FtsZ assembly by light scattering and determination of the role of divalent metal cations. Journal of Bacteriology.

[bib53] Nguyen LT, Oikonomou CM, Jensen GJ (2021). Simulations of proposed mechanisms of FtsZ-driven cell constriction. Journal of Bacteriology.

[bib54] Nogales E, Downing KH, Amos LA, Löwe J (1998). Tubulin and FtsZ form a distinct family of GTPases. Nature Structural Biology.

[bib55] Ortiz C, Natale P, Cueto L, Vicente M (2016). The keepers of the ring: regulators of FtsZ assembly. FEMS Microbiology Reviews.

[bib56] Pazos M, Natale P, Margolin W, Vicente M (2013). Interactions among the early *Escherichia coli* divisome proteins revealed by bimolecular fluorescence complementation. Environmental Microbiology.

[bib57] Pettersen EF, Goddard TD, Huang CC, Couch GS, Greenblatt DM, Meng EC, Ferrin TE (2004). UCSF Chimera--a visualization system for exploratory research and analysis. Journal of Computational Chemistry.

[bib58] Peulen TO, Hemmen K (2024). GitHub.

[bib59] Popp D, Iwasa M, Narita A, Erickson HP, Maéda Y (2009). FtsZ condensates: an in vitro electron microscopy study. Biopolymers.

[bib60] Ramirez-Diaz DA, García-Soriano DA, Raso A, Mücksch J, Feingold M, Rivas G, Schwille P (2018). Treadmilling analysis reveals new insights into dynamic FtsZ ring architecture. PLOS Biology.

[bib61] Reija B, Monterroso B, Jiménez M, Vicente M, Rivas G, Zorrilla S (2011). Development of a homogeneous fluorescence anisotropy assay to monitor and measure FtsZ assembly in solution. Analytical Biochemistry.

[bib62] Rivas G, López A, Mingorance J, Ferrándiz MJ, Zorrilla S, Minton AP, Vicente M, Andreu JM (2000). Magnesium-induced linear self-association of the FtsZ bacterial cell division protein monomer: the primary steps for FtsZ assembly. The Journal of Biological Chemistry.

[bib63] Roach EJ, Wroblewski C, Seidel L, Berezuk AM, Brewer D, Kimber MS, Khursigara CM (2016). Structure and mutational analyses of *Escherichia coli* ZapD reveal charged residues involved in FtsZ filament bundling. Journal of Bacteriology.

[bib64] Rowlett VW, Margolin W (2014). 3D-SIM super-resolution of FtsZ and its membrane tethers in *Escherichia coli* cells. Biophysical Journal.

[bib65] Schuck P (2000). Size-distribution analysis of macromolecules by sedimentation velocity ultracentrifugation and lamm equation modeling. Biophysical Journal.

[bib66] Schuck P (2003). On the analysis of protein self-association by sedimentation velocity analytical ultracentrifugation. Analytical Biochemistry.

[bib67] Schumacher MA, Zeng W, Huang KH, Tchorzewski L, Janakiraman A (2016). Structural and functional analyses reveal insights into the molecular properties of the *Escherichia coli* Z ring stabilizing protein, ZapC. The Journal of Biological Chemistry.

[bib68] Schumacher MA, Huang KH, Zeng W, Janakiraman A (2017). Structure of the Z ring-associated protein, ZapD, bound to the C-terminal domain of the tubulin-like protein, FtsZ, suggests mechanism of Z ring stabilization through FtsZ cross-linking. The Journal of Biological Chemistry.

[bib69] Schur FKM, Obr M, Hagen WJH, Wan W, Jakobi AJ, Kirkpatrick JM, Sachse C, Kräusslich H-G, Briggs JAG (2016). An atomic model of HIV-1 capsid-SP1 reveals structures regulating assembly and maturation. Science.

[bib70] Söderström B, Daley DO (2017). The bacterial divisome: more than a ring?. Current Genetics.

[bib71] Squyres GR, Holmes MJ, Barger SR, Pennycook BR, Ryan J, Yan VT, Garner EC (2021). Single-molecule imaging reveals that Z-ring condensation is essential for cell division in *Bacillus subtilis*. Nature Microbiology.

[bib72] Stricker J, Maddox P, Salmon ED, Erickson HP (2002). Rapid assembly dynamics of the *Escherichia coli* FtsZ-ring demonstrated by fluorescence recovery after photobleaching. PNAS.

[bib73] Sundararajan K, Goley ED (2017). The intrinsically disordered C-terminal linker of FtsZ regulates protofilament dynamics and superstructure *in vitro*. The Journal of Biological Chemistry.

[bib74] Sundararajan K, Vecchiarelli A, Mizuuchi K, Goley ED (2018). Species- and C-terminal linker-dependent variations in the dynamic behavior of FtsZ on membranes in vitro. Molecular Microbiology.

[bib75] Szwedziak P, Wang Q, Bharat TAMM, Tsim M, Löwe J (2014). Architecture of the ring formed by the tubulin homologue FtsZ in bacterial cell division. eLife.

[bib76] Szwedziak P, Ghosal D (2017). FtsZ-ring architecture and its control by MinCD. Sub-Cellular Biochemistry.

[bib77] Tonthat NK, Arold ST, Pickering BF, Van Dyke MW, Liang S, Lu Y, Beuria TK, Margolin W, Schumacher MA (2011). Molecular mechanism by which the nucleoid occlusion factor, SlmA, keeps cytokinesis in check. The EMBO Journal.

[bib78] Vélez M (2022). How does the spatial confinement of ftsz to a membrane surface affect its polymerization properties and function?. Frontiers in Microbiology.

[bib79] Wagstaff JM, Tsim M, Oliva MA, García-Sanchez A, Kureisaite-Ciziene D, Andreu JM, Löwe J (2017). A polymerization-associated structural switch in FtsZ that enables treadmilling of model filaments. mBio.

[bib80] Wan W (2024). GitHub.

[bib81] Wang X, Huang J, Mukherjee A, Cao C, Lutkenhaus J (1997). Analysis of the interaction of FtsZ with itself, GTP, and FtsA. Journal of Bacteriology.

[bib82] Wang N, Bian L, Ma X, Meng Y, Chen CS, Rahman MU, Zhang T, Li Z, Wang P, Chen Y (2019). Assembly properties of the bacterial tubulin homolog FtsZ from the cyanobacterium *Synechocystis* sp. PCC 6803. The Journal of Biological Chemistry.

[bib83] Wang M, Fang C, Ma B, Luo X, Hou Z (2020). Regulation of cytokinesis: FtsZ and its accessory proteins. Current Genetics.

[bib84] Whitley KD, Jukes C, Tregidgo N, Karinou E, Almada P, Cesbron Y, Henriques R, Dekker C, Holden S (2021). FtsZ treadmilling is essential for Z-ring condensation and septal constriction initiation in *Bacillus subtilis* cell division. Nature Communications.

[bib85] Yang X, Lyu Z, Miguel A, McQuillen R, Huang KC, Xiao J (2017). GTPase activity-coupled treadmilling of the bacterial tubulin FtsZ organizes septal cell wall synthesis. Science.

[bib86] Zheng SQ, Palovcak E, Armache J-P, Verba KA, Cheng Y, Agard DA (2017). MotionCor2: anisotropic correction of beam-induced motion for improved cryo-electron microscopy. Nature Methods.

