# Supplementary figures and images for: Crosslinking by ZapD drives the assembly of short FtsZ filaments into toroidal structures in solution
Source: eLife. 2025 Sep 15;13:RP95557. doi: 10.7554/eLife.95557 (PMC12435895; doi:10.7554/eLife.95557)

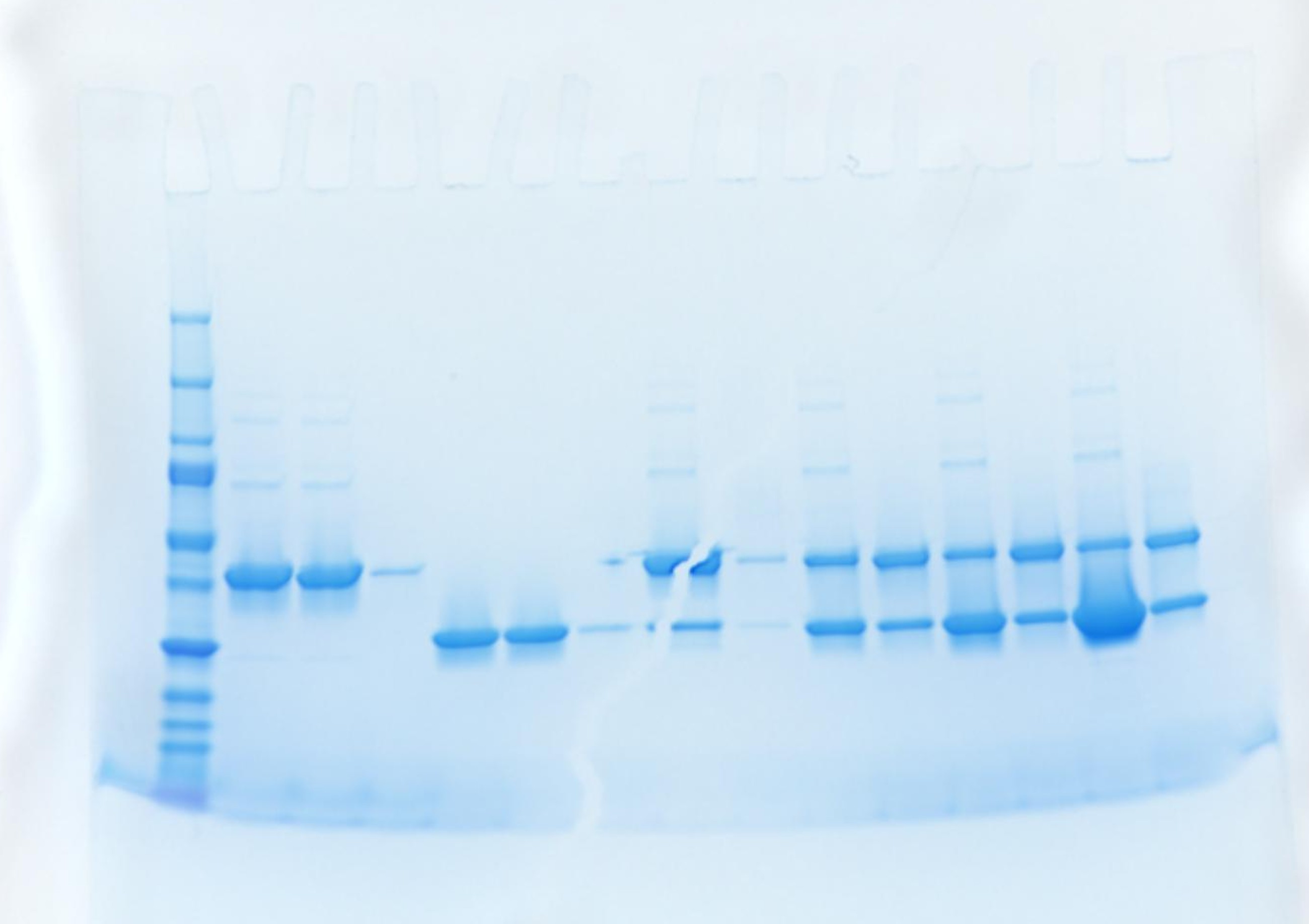

Supplement: Figure 1—figure supplement 5—source data 1. [file elife-95557-fig1-figsupp5-data1.zip › Gel5.jpg]

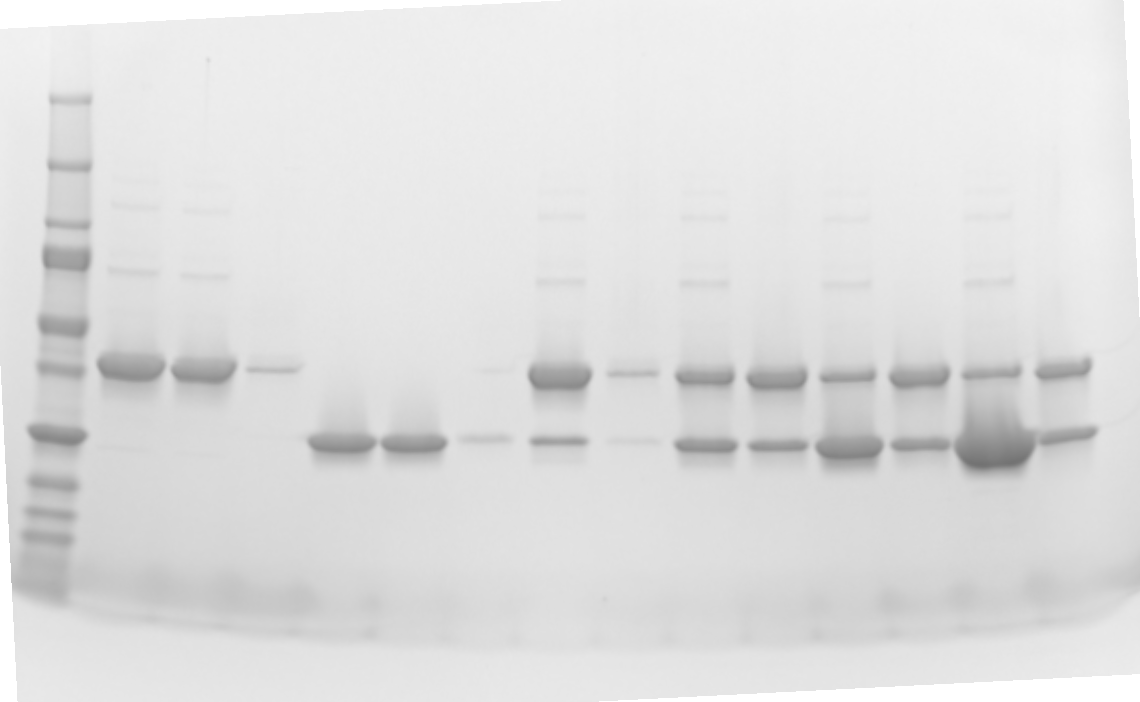

Supplement: Figure 1—figure supplement 5—source data 1. [file elife-95557-fig1-figsupp5-data1.zip › Gel1.tif]

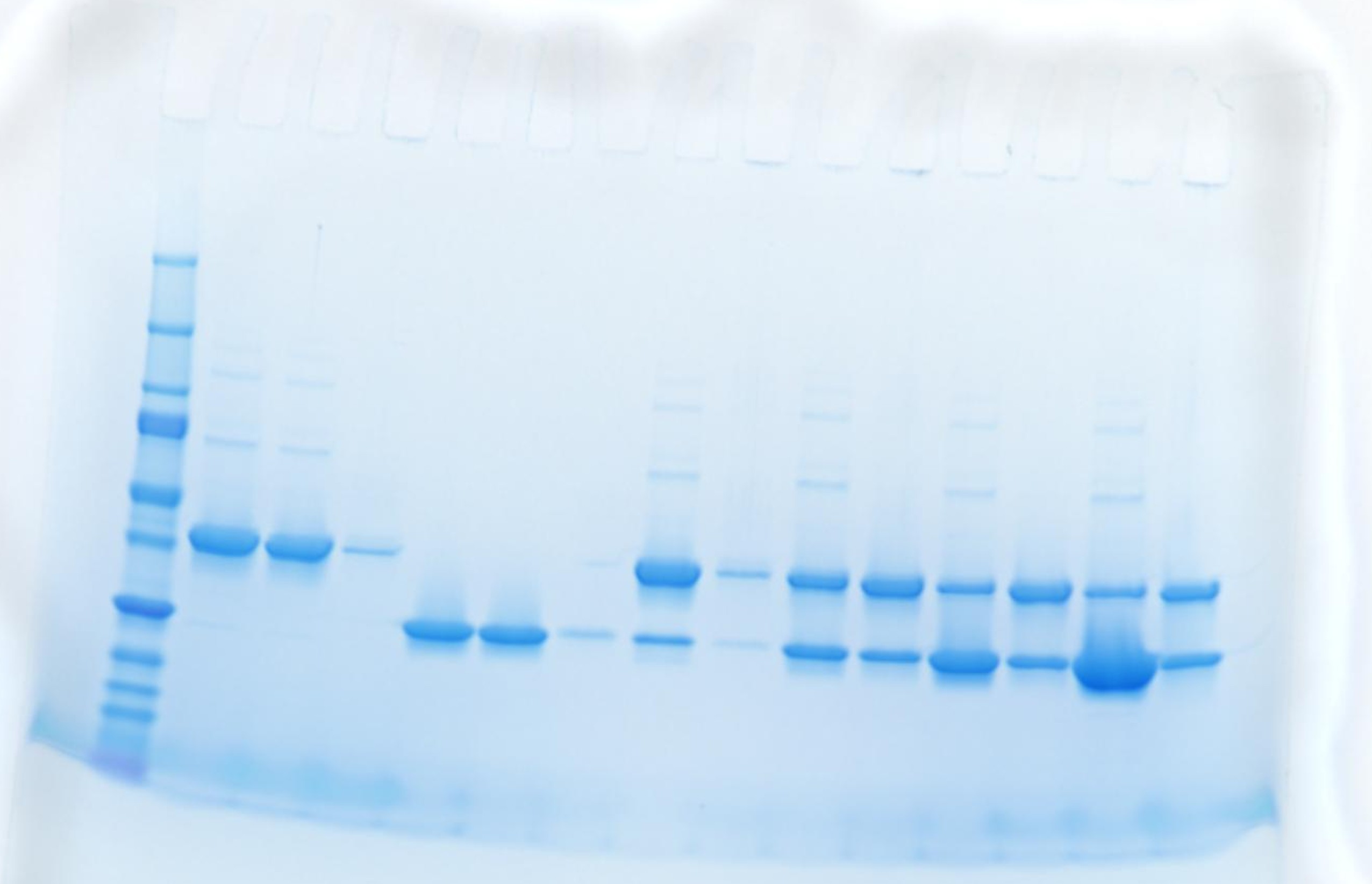

Supplement: Figure 1—figure supplement 5—source data 1. [file elife-95557-fig1-figsupp5-data1.zip › Gel1-2.jpg]

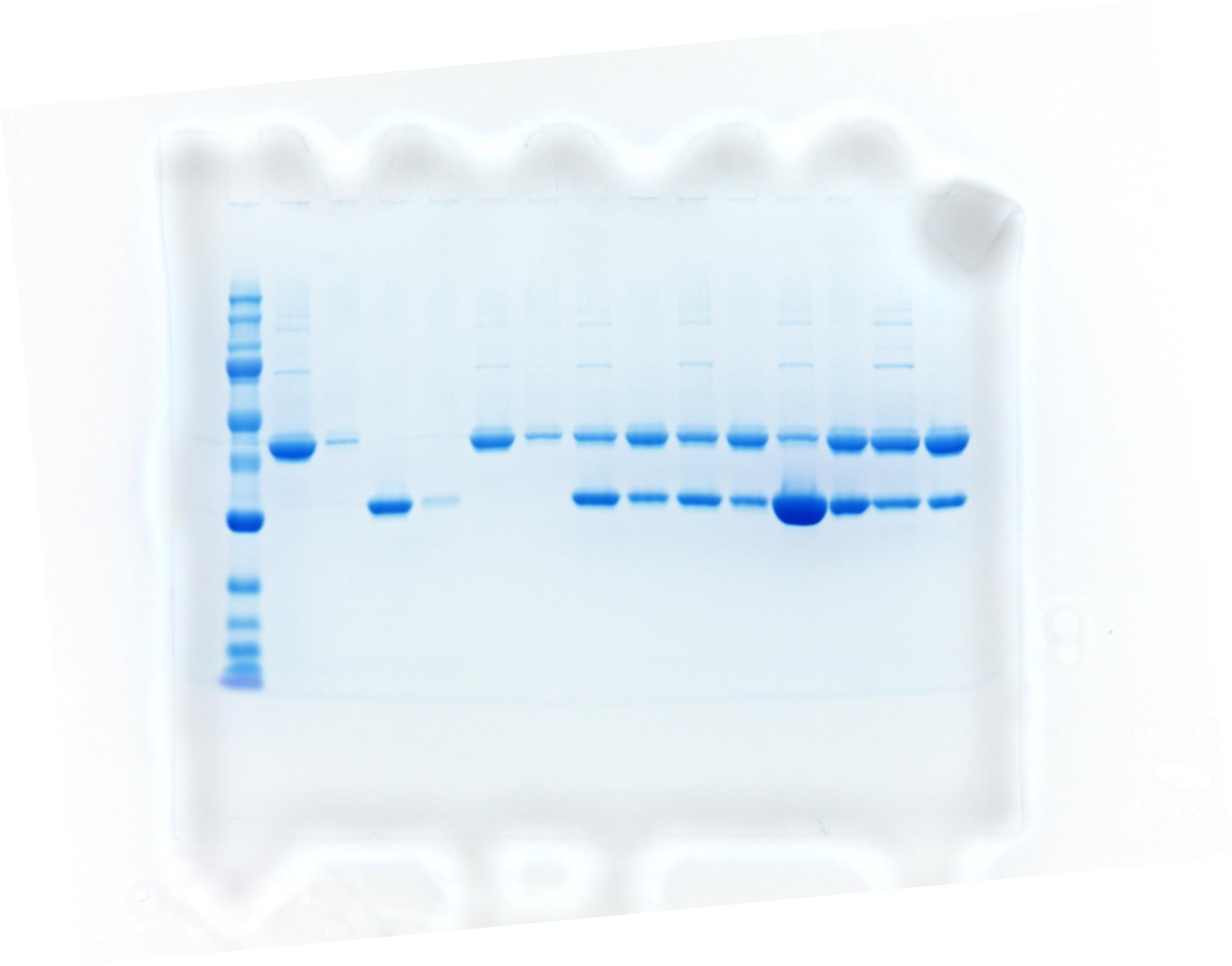

Supplement: Figure 1—figure supplement 5—source data 1. [file elife-95557-fig1-figsupp5-data1.zip › Gel2.jpg]

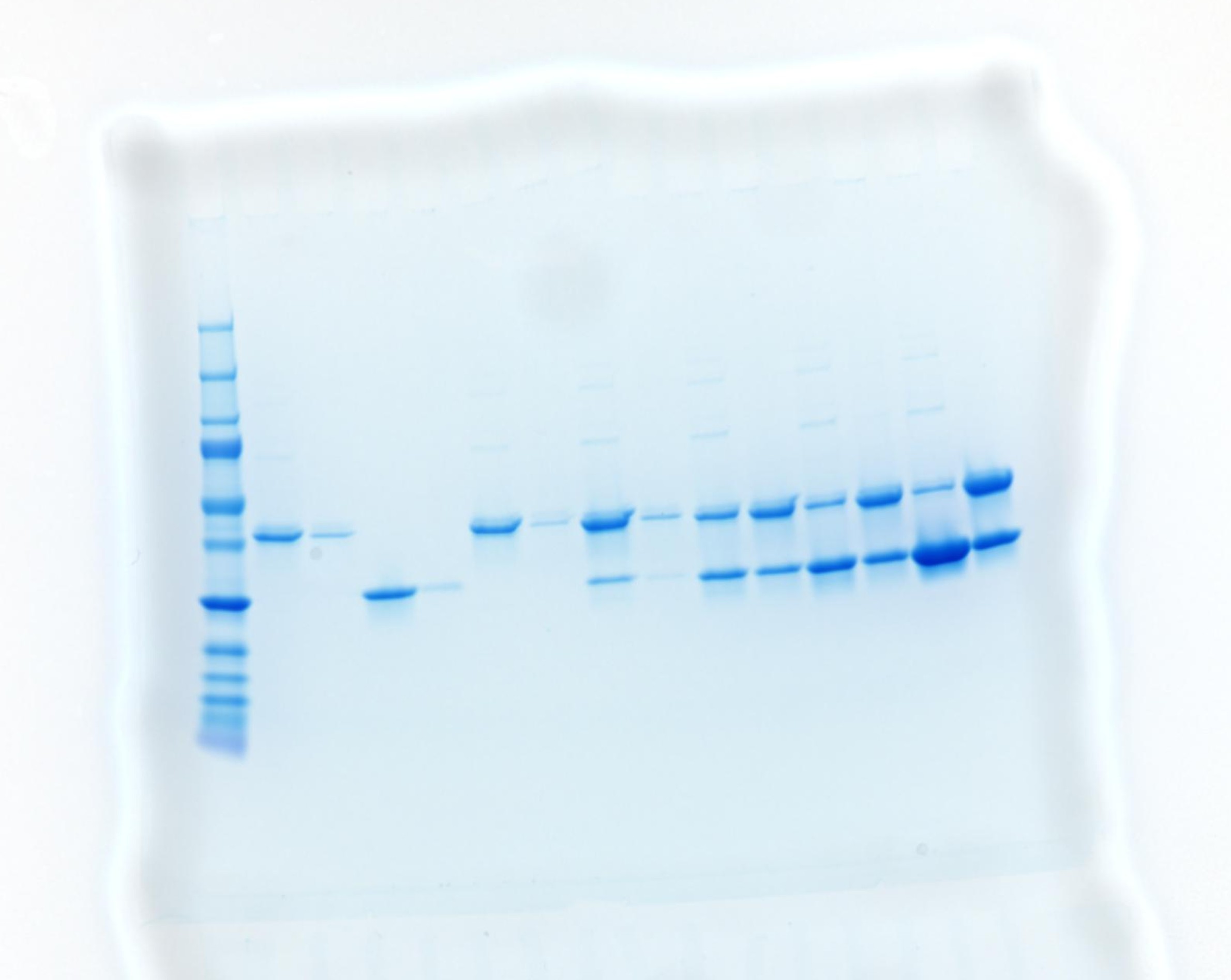

Supplement: Figure 1—figure supplement 5—source data 1. [file elife-95557-fig1-figsupp5-data1.zip › Gel3.jpg]

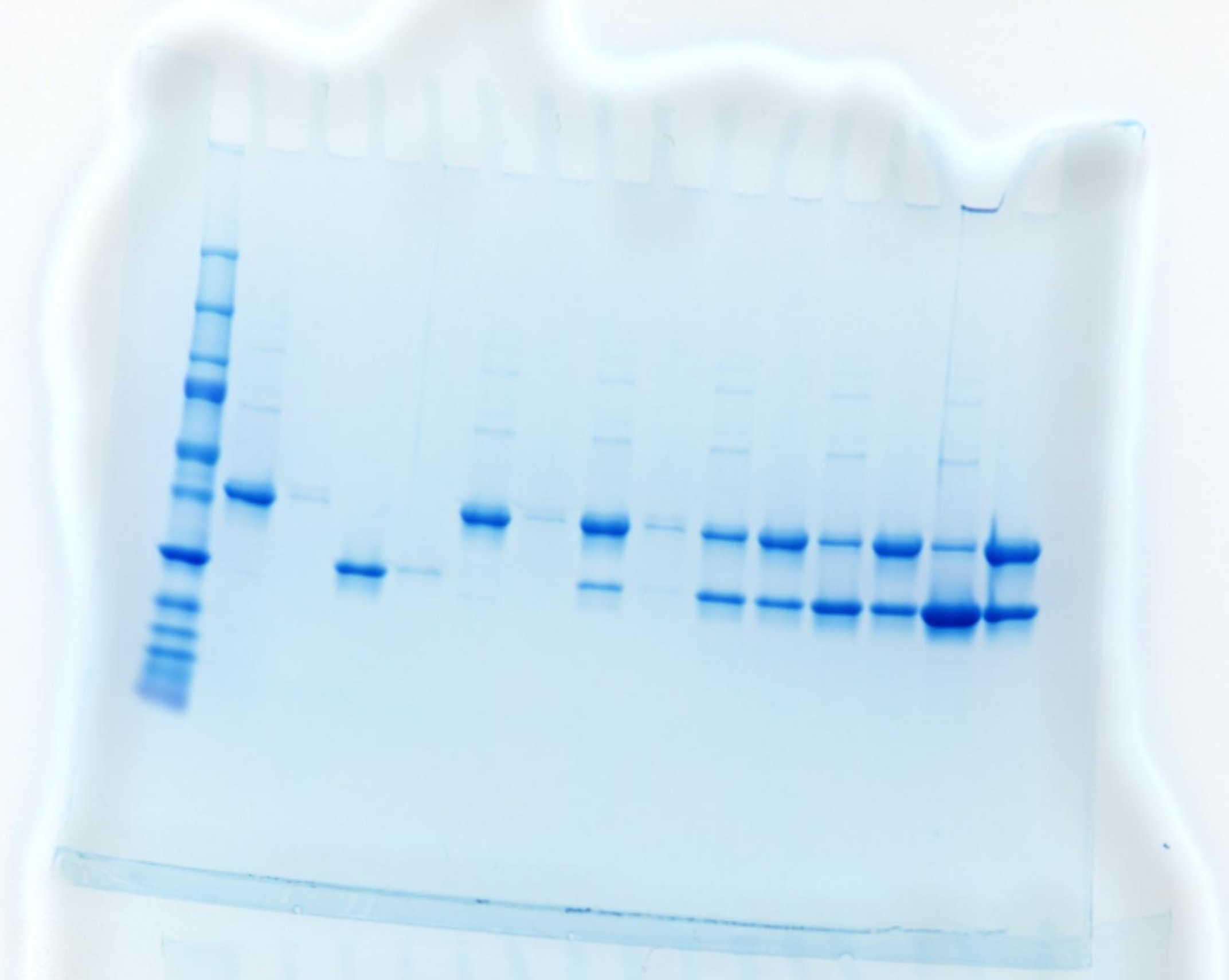

Supplement: Figure 1—figure supplement 5—source data 1. [file elife-95557-fig1-figsupp5-data1.zip › Gel4.jpg]
