# Supplementary material for: Crosslinking by ZapD drives the assembly of short FtsZ filaments into toroidal structures in solution
Source: eLife. 2025 Sep 15;13:RP95557. doi: 10.7554/eLife.95557 (PMC12435895; doi:10.7554/eLife.95557)
Supplement: Figure 1—figure supplement 5—source data 2. [file elife-95557-fig1-figsupp5-data2.zip › Figure 1ΓÇöfigure supplement 5ΓÇösource data 2.pdf]

1

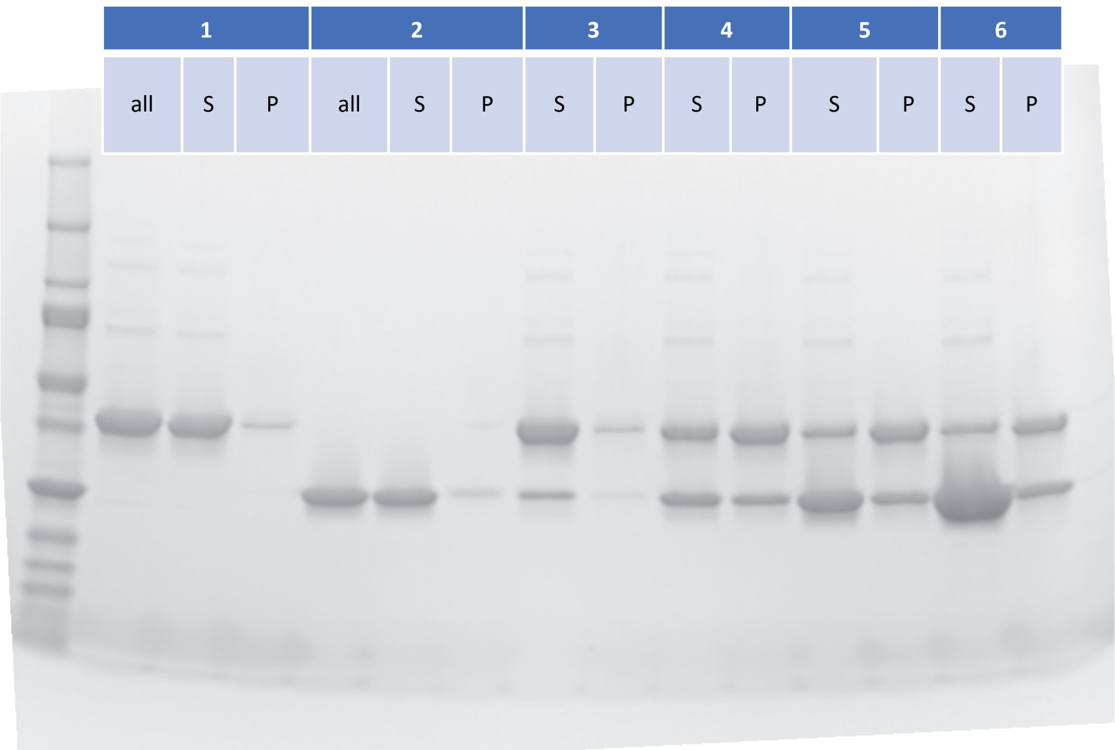

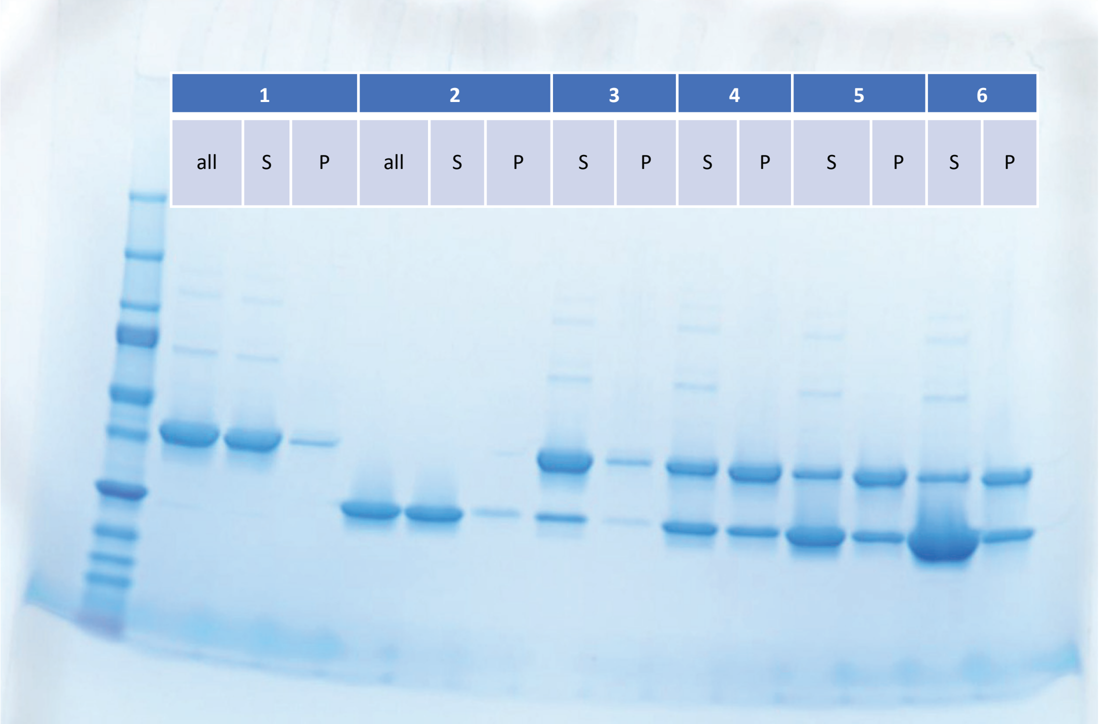

	1	2	3	4	5	6
**[stock]**	FtsZ cntrl	ZapD cntrl	FtsZ+ ZapD	FtsZ + ZapD	FtsZ + ZapD	FtsZ + ZapD
**FtsZ (uM)**	5	0	5	5	5	10
**ZapD (uM)**	0	5	1	5	30	10

P= Pellet
S= Supernatant
All= All sample without centrifugation

2

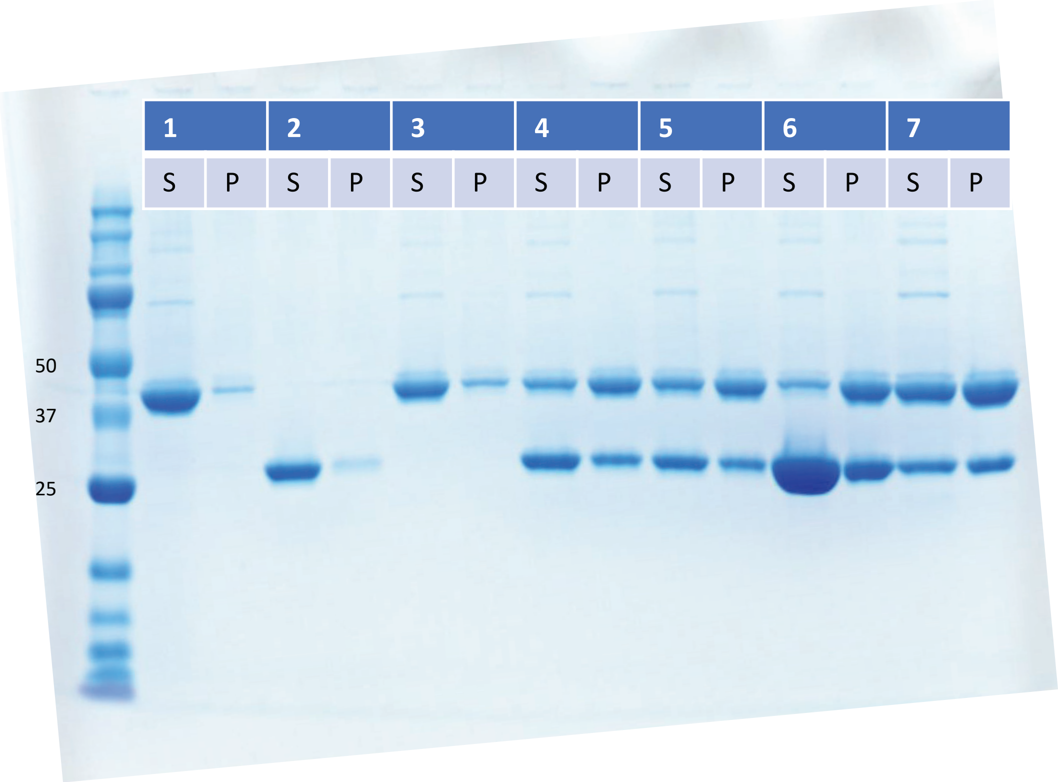

	1	2	3	4	5	6	7
**[stock]**	FtsZ cntrl	ZapD cntrl	FtsZ cntrl GDP	FtsZ+ ZapD	FtsZ + ZapD	FtsZ + ZapD	FtsZ + ZapD
**FtsZ (uM)**	5	0	5	5	5	5	10
**ZapD (uM)**	0	5	0	1	5	30	10

3

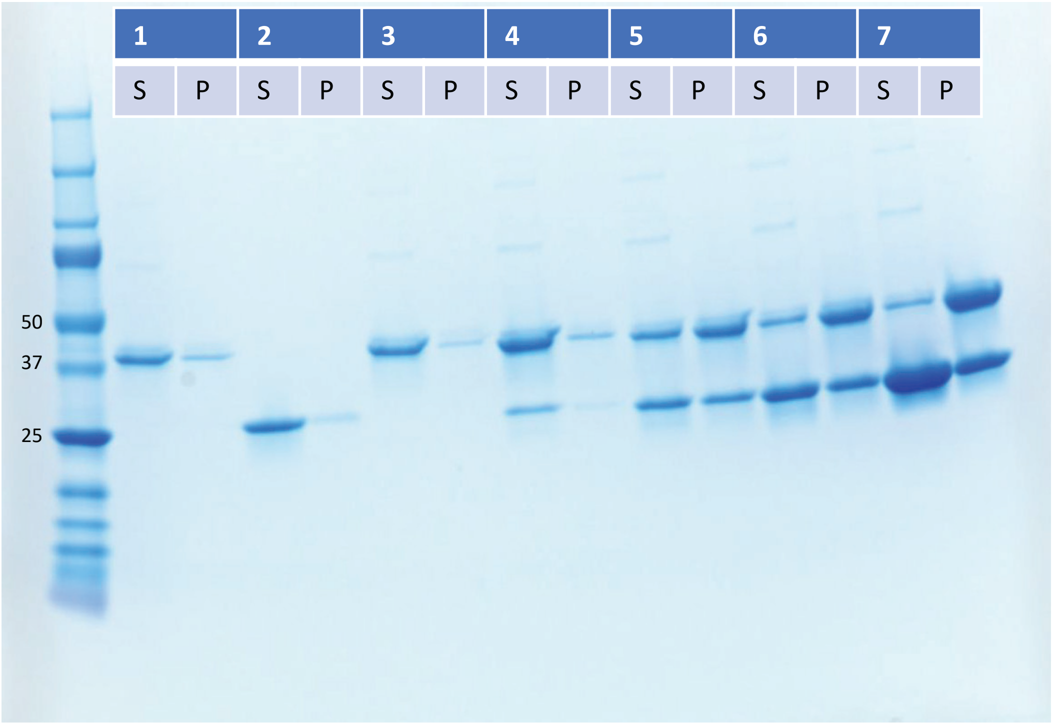

	1	2	3	4	5	6	7
**[stock]**	FtsZ cntrl	ZapD cntrl	FtsZ cntrl GDP	FtsZ+ ZapD	FtsZ + ZapD	FtsZ + ZapD	FtsZ + ZapD
**FtsZ (uM)**	5	0	5	5	5	5	5
**ZapD (uM)**	0	5	0	1	5	10	30

4

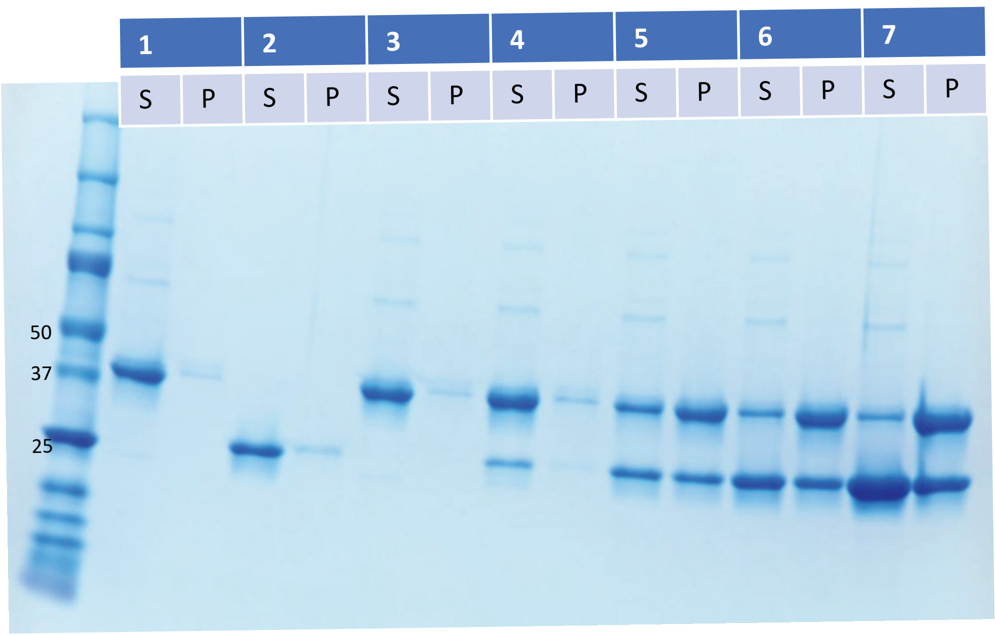

	1	2	3	4	5	6	7
**[stock]**	FtsZ cntrl	ZapD cntrl	FtsZ cntrl GDP	FtsZ+ ZapD	FtsZ + ZapD	FtsZ + ZapD	FtsZ + ZapD
**FtsZ (uM)**	5	0	5	5	5	5	5
**ZapD (uM)**	0	5	0	1	5	10	30

5

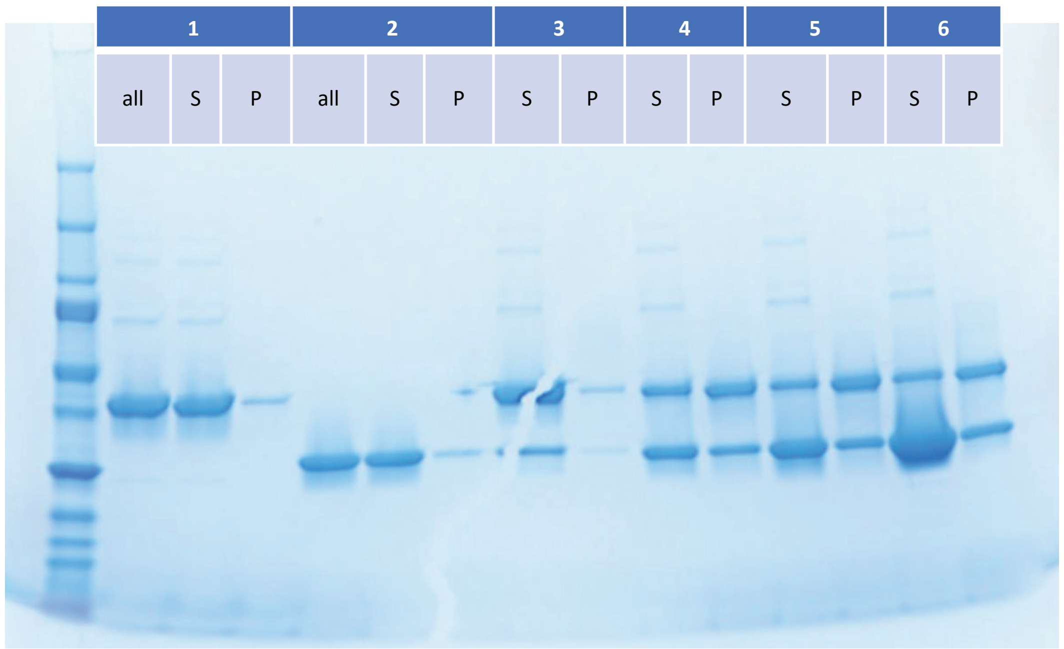

	1	2	3	4	5	6
**[stock]**	FtsZ cntrl	ZapD cntrl	FtsZ+ ZapD	FtsZ + ZapD	FtsZ + ZapD	FtsZ + ZapD
**FtsZ (uM)**	5	0	5	5	5	10
**ZapD (uM)**	0	5	1	5	30	10
